# Identification and experimental validation of CCL26 as a core comorbid gene and biomarker for atopic dermatitis and allergic asthma via machine learning and multi-omics analysis

**DOI:** 10.3389/fimmu.2026.1936742

**Published:** 2026-09-09

**Authors:** Yunfan Gu, Shilei Shi, Yuqing Rao, Shuang Zhang, Jiaqi Zhang, Weiming Zhang, Xianyu Zeng

**Affiliations:** 1 School of Traditional Chinese Medicine, Hubei University of Chinese Medicine, Wuhan, China; 2 First Clinical College, Hubei University of Chinese Medicine, Wuhan, China; 3 Department of Dermatology, Traditional Chinese and Western Medicine Hospital of Wuhan, Tongji Medical College, Huazhong University of Science and Technology, Wuhan, Hubei, China

**Keywords:** allergic asthma, atopic dermatitis, CCL26, chemotactic cytokines, machine learning, single-cell transcriptomics, spatial transcriptomics

## Abstract

**Background:**

Atopic dermatitis (AD) and allergic asthma (AA) frequently co-occur and are a core component of the “atopic march.” However, the shared molecular mechanisms linking these conditions, as well as the core comorbid genes with diagnostic value, remain poorly understood.

**Methods:**

This study integrated differential expression analysis and weighted gene co-expression network analysis (WGCNA) of bulk RNA sequencing data from AD and AA to identify comorbidity-associated genes. Diagnostic models were built using 8 feature selection algorithms and 175 machine-learning combinations, and then validated across multiple independent external cohorts. Single-cell transcriptomic data from AD and AA were integrated to identify pathogenic cell subclusters using the Scissor, scAB, and DEGAS algorithms and to examine the distribution of comorbid genes. Their functional mechanisms were clarified through virtual gene knockouts (Geneformer, CellOracle, and scTenifoldKnk) and cell-cell communication analyses (CellChat and MultiNicheNet), and were validated *in situ* using 10x Visium (AD) and Xenium (AA) spatial transcriptomics data. Finally, Candidate hub genes were validated *in vitro* using IL-4 and IL-13-stimulated tissue-specific cellular models.

**Results:**

Following differential expression analysis, WGCNA, and machine-learning-based feature selection, CCL26 showed consistent and significant upregulation across all cohorts. A diagnostic model based on CCL26 performed well in the external validation cohort. Single-cell analysis revealed that CCL26 is specifically enriched in pathogenic cell subsets, including inflammatory vascular smooth muscle cells and fibroblasts in AD skin, as well as basal- and secretory-lineage epithelial cells in AA airways. Virtual gene knockout revealed that CCL26 is involved in regulating tissue remodeling and inflammatory pathways. Cell-to-cell communication and spatial transcriptomic analyses further confirmed that CCL26 mediates communication between pathogenic cell subsets and Th2 cells via the CCR3 receptor, leading to the formation of self-amplifying inflammatory microenvironments in the skin and airways. *In vitro* experiments confirmed that CCL26 expression was significantly elevated in IL-4 and IL-13-stimulated HDF and BEAS-2B cells.

**Conclusion:**

This study identifies CCL26 as a core comorbid gene and a potential diagnostic biomarker linking AD and AA. Furthermore, it elucidates the molecular mechanism by which CCL26 drives skin-airway inflammation via the CCL26-CCR3 axis, offering a novel perspective on atopic comorbidities.

## 1 Introduction

Atopic dermatitis (AD) and asthma are two common chronic inflammatory diseases that impose a heavy medical and socioeconomic burden globally. According to the 2019 Global Burden of Disease study estimates, asthma affects approximately 262 million people worldwide and leads to about 455,000 deaths annually (1); the prevalence of AD is as high as one-fifth during childhood, and the adult prevalence also remains at 3%–5% (2). Although the number of affected individuals for both diseases continues to rise due to population growth, the age-standardized prevalence rates between 1990 and 2019 have decreased, and both show a bimodal pattern, peaking in childhood (ages 5–9 years) and adulthood (3). More importantly, there is a clear comorbid relationship between the two, termed the “atopic march,” which typically begins with AD in early childhood and subsequently progresses to asthma and/or allergic rhinitis (4). A meta-analysis incorporating 39 prospective cohort studies with a total of 458810 participants showed that the relative risk of AD patients developing asthma is 2.16, and the risk increases in a dose-dependent manner with the duration and severity of AD (5); this dose-response relationship was also confirmed in a United Kingdom electronic health record cohort of approximately 5.5 million patients, and the severity of AD is also directly associated with an elevated risk of asthma exacerbations and hospitalizations (6). Within the AD population, the comorbid prevalence rates of asthma, rhinitis, or their coexistence are 40.5%, 25.7%, and 14.2%, respectively, and the odds of patients developing another atopic disease are 3 to 4 times higher than in the non-AD population (2).

Notably, asthma is a highly heterogeneous clinical syndrome that can be classified into two major endophenotypes, allergic and non-allergic, based on serum-specific IgE levels and skin prick test results. The impact of AD on the two is not uniform; a 40-year prospective cohort study showed that childhood eczema and rhinitis can predict allergic asthma (AA) in adulthood but not non-allergic asthma (7). This suggests that the causal chain of the “atopic march” is essentially driven by IgE-mediated sensitization. Therefore, the portion of the AD-asthma comorbidity with true mechanistic homology should focus on AA. The present study thus takes the comorbidity of AD and AA as the core subject of investigation.

To elucidate the pathophysiological foundation behind the AD and AA comorbidity, the “outside-inside-outside” hypothesis proposed by Peter M Elias and colleagues takes the disruption of the AD skin barrier as the initiating event, triggering immune dysregulation that drives systemic allergic sensitization and extending “from the outside in” to affect the airways, ultimately leading to AA (8). Although this systemic Th2 activation state conceptually links the allergic inflammatory manifestations of the skin and the respiratory system, the molecular mechanisms that span the two tissues remain unclear. Moreover, previous biomarker studies have mostly been confined to the respective disease backgrounds of AD or AA in isolation, and systematic transcriptomic research targeting the cross-phenotype of “AD-AA comorbidity” can further the understanding of atopic comorbidity and facilitate the search for comorbid biomarkers with diagnostic value.

**Figure 1:**
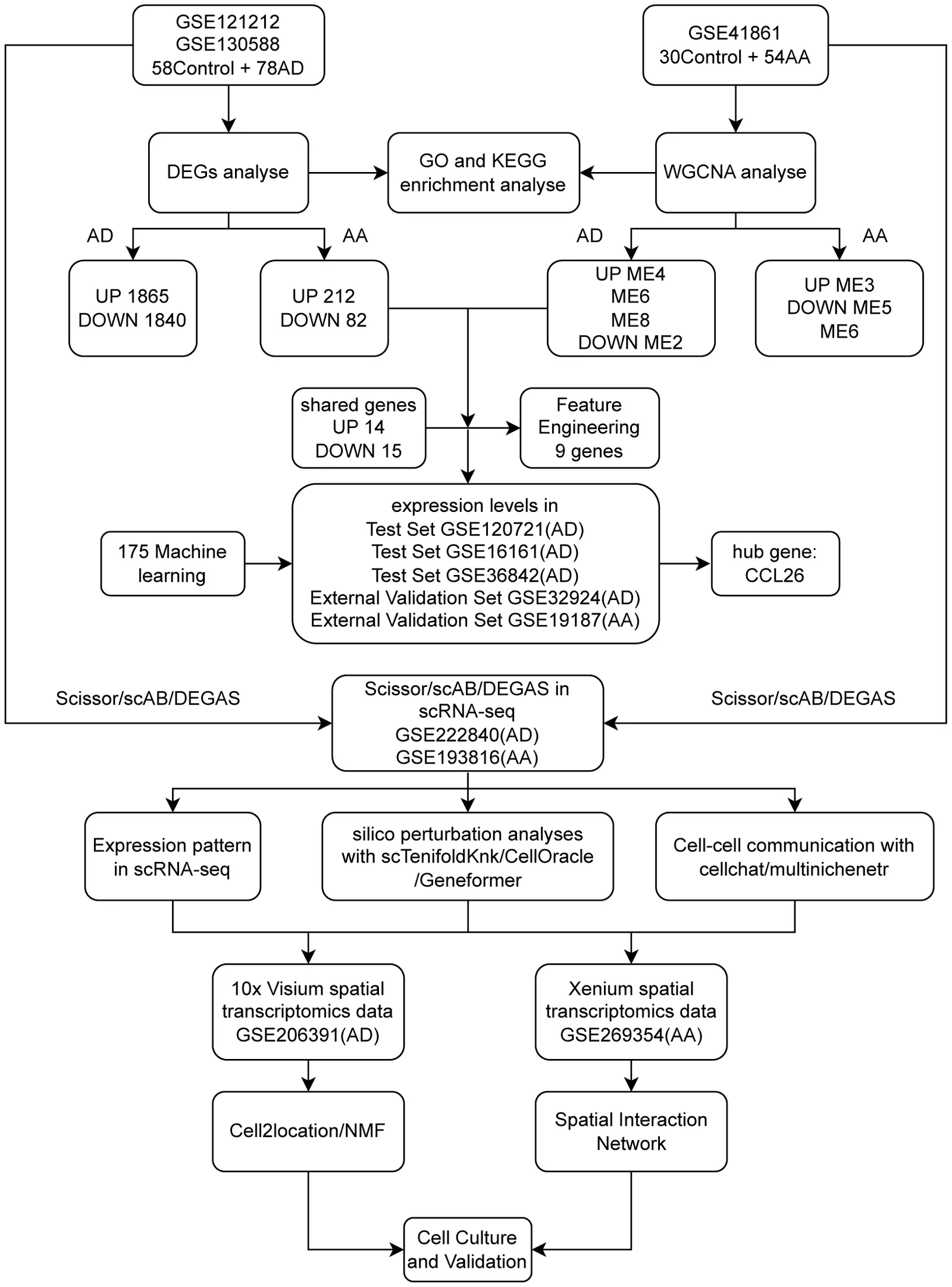
Flow chart of the study.

In this study, we performed an integrative, multi-stage transcriptomic analysis to identify and validate a core comorbid gene linking AD and AA. First, using bulk RNA sequencing datasets for AD and AA obtained from the Gene Expression Omnibus (GEO), we combined differential expression analysis (DEA) and weighted gene co-expression network analysis (WGCNA) with machine learning-based feature engineering to screen candidate core comorbid genes, which were then integrated into multiple algorithmic combinations to construct a comorbidity diagnostic model. The model’s performance was assessed on an internal test set and further confirmed in independent external validation cohorts. Second, we analyzed single-cell transcriptomic data from AD and AA using the Scissor, scAB, and DEGAS algorithms to identify disease-associated pathogenic cell subgroups, characterize the expression patterns of the comorbid gene within these subgroups, and elucidate its functional relevance through cell-cell communication analysis and virtual gene knockouts. Third, we used spatial transcriptomic data generated by 10x Visium and Xenium to validate these findings *in situ*, revealing spatial co-occurrence between the target cell subsets that highly express the comorbid gene and effector cells. Finally, we validated the expression of the comorbid gene in tissue-specific cellular models. The overall workflow of this study is illustrated in **Figure 1**.

## 2 Materials and methods

### 2.1 Data source and processes

Publicly available transcriptomic datasets for AD and AA were obtained from the GEO database. For the discovery stage, bulk RNA sequencing datasets were used, comprising two AD cohorts (GSE121212, GSE130588) and one AA cohort (GSE41861). Three additional AD datasets (GSE120721, GSE16161, GSE36842) served as an internal testing set, while GSE32924 (AD) and GSE19187 (AA) were used as independent external validation cohorts. For single-cell transcriptomic analyses, one AD dataset (GSE222840) and one AA dataset (GSE193816) were included. For spatial transcriptomic validation, a 10x Visium dataset for AD (GSE206391) and a Xenium dataset for AA (GSE269354) were used. **Supplementary Table 1** summarizes comprehensive details for all included datasets, including accession numbers, specimen/tissue origins, sample sizes (control/disease cohorts), sequencing platforms, and technologies.

### 2.2 Differential expression analysis and WGCNA

To achieve a sufficient sample size and enhance statistical power, two independent AD datasets from different transcriptomic platforms (GSE121212 based on RNA-seq and GSE130588 based on microarray) were integrated. The RNA-seq data were pre-transformed into continuous log2-CPM values using the cpm function within the ‘edgeR’ package in R (version 4.4.2) to ensure cross-platform scale compatibility. Inherent batch effects and platform leverage between the datasets were subsequently eliminated using the ComBat algorithm via the “sva” R package. Principal Component Analysis (PCA) was conducted before and after integration to confirm the removal of batch effects. For the AA dataset (GSE41861), which was generated on a single microarray platform, raw intensity values were directly log2-transformed and normalized without requiring cross-platform integration.

DEA was performed on the batch-corrected, integrated AD expression matrix and the normalized AA expression matrix separately using the “limma” R package to identify genes differentially expressed between the disease and control groups, where for the AD analysis, platform source was additionally incorporated into the linear model as a blocking factor to intercept any residual batch variance. Differentially expressed genes (DEGs) for AD and AA were defined using a threshold of |log_2_ fold change (FC)| > 0.585 and adjusted *P* < 0.05. The expression and distribution characteristics of DEGs were visualized using the “ggplot2” and “pheatmap” R packages.

Concurrently, WGCNA was performed to identify gene co-expression modules with high biological significance using AD and AA expression matrices (9). Under the scale-free topology criterion, the soft-thresholding powers (β) were selected using the “WGCNA” R package, ensuring that the scale-free topology model fit index (R^2^) cleared the standard threshold of 0.9 for both AD and AA datasets. Co-expression modules were identified using the dynamic tree cut method (minimum size = 200) based on the topological overlap matrix, followed by the fusion of closed modules with a merge threshold of 0.1. Finally, modules correlated with the traits were selected based on Pearson correlation coefficients between module eigengenes (MEs) and sample traits. Modules with a statistically significant correlation (*P* < 0.05) and an absolute correlation coefficient greater than 0.5 (or those closest to 0.5) were chosen as core modules. To visualize the module gene network, the WGCNA topology overlap matrix was exported, and a force-directed network diagram was constructed using the “graph” R package.

Gene Ontology (GO) and Kyoto Encyclopedia of Genes and Genomes (KEGG) analyses were performed on DEGs and MEs using the “clusterProfiler” R package, and Venn diagrams were used to identify core candidate genes for comorbidities to support feature engineering.

### 2.3 Feature engineering and comorbidity diagnostic model construction

A feature engineering pipeline comprising eight robust feature selection algorithms (Random Forest (RF), Gradient Boosting Machine (GBM), Lasso, CatBoost, LightGBM, decision trees, Partial Least Squares (PLS), and discriminant models) was established to screen candidate genes. Features selected by at least seven algorithms were defined as core comorbidity features for use in building the comorbidity diagnostic model.

To mitigate overfitting, we combined 15 basic machine learning algorithms (Lasso, Ridge, Elastic Net, Stepwise GLM (Stepglm), Support Vector Machine (SVM), Linear Discriminant Analysis (LDA), Quadratic Discriminant Analysis (QDA), glmBoost, Partial Least Squares Regression Generalized Linear Model (plsRglm), RF, GBM, XGBoost, Naïve Bayes, K-Nearest Neighbors (KNN), and AdaBoost) into 175 modeling strategies.

The model was trained on a combined AD dataset (GSE121212 and GSE130588), tested on GSE120721, GSE16161, and GSE36842, and independently validated using external AD and AA cohorts (GSE32924, and GSE19187). The optimal model was selected based on the highest average area under the receiver operating characteristic curve (ROC) across the training and testing sets.

### 2.4 scRNA-seq data quality control and annotation

Further investigation of the distribution and pathogenic mechanisms of core comorbid genes in scRNA-seq datasets for AD (GSE222840) and AA (GSE193816). For the AD dataset, potential double cells were removed using “Scrublet” (v0.2.3) in Python 3.10. Utilizing the “Seurat” (v5.3.1) R package, cells were filtered based on the following criteria: gene counts and total transcripts between 200 and the 97th percentile, and mitochondrial content < 15%. Following normalization and log-transformation, the top 2000 highly variable genes were identified for PCA. Batch effects across samples were integrated using Harmony (v1.2.3). Ambient RNA contamination was purged using the “decontX” (v1.4.1) with a contamination threshold < 20%. The AA dataset, which had already undergone stringent quality control (including the removal of low-quality cells with < 500 detected unique genes or > 30% mitochondrial content, as well as doublet filtration via biologically informed marker exclusions and Scrublet) (10), was converted from h5ad to Seurat using the “scBridge” R package. Finally, the FindNeighbors and FindClusters functions were applied to identify cell clusters, and cell types were annotated by consulting the CellMarker database and the existing literature (10, 11).

### 2.5 Phenotype-associated pathogenic cell subset identification using Scissor, scAB, and DEGAS

To identify cell subsets contributing to disease onset, we used two complementary methods, Scissor (based on regression models and correlation analysis) (12) and scAB (a knowledge- and graph-guided matrix factorization model) (13) to integrate clinical phenotypes with bulk RNA-seq and scRNA-seq data. Using the R packages Scissor and scAB to extract clinical phenotypes from AD (GSE121212/GSE130588) and AA (GSE41861), we mapped cell-level clinical correlations onto the corresponding scRNA-seq matrices to identify core pathogenic cell subpopulations Scissor+/scAB+. Additionally, to robustly map patient-level disease phenotypes onto individual cells via multi-trait transfer learning, the deep learning-based framework DEGAS (Diagnostic Evidence GAuge of Single cells) (14) was implemented using the “DEGAS” R packages (v1.0.0), linked to a Python environment. Highly variable genes from the AD and AA single-cell datasets were intersected with the bulk RNA expression matrices of the AD and AA discovery cohorts, respectively, to obtain common gene sets. The DEGAS preprocess Counts function was used to normalize the matrices, and the disease labels in the bulk RNA data, along with the single-cell cluster cell types, were converted into one-hot encodings. The DEGAS model, featuring a dense neural network, was trained using the runCCMTLBag function, which included a 3-layer network architecture and 5 resampling repetitions. Finally, the predClassBag function predicted the cell-level disease-associated probabilities, and the association scores were mapped back into the single-cell metadata slots to cross-validate the pathogenic cell subpopulations identified by Scissor and scAB.

Subsequently, to address the sparsity and high variability of single-cell data, we performed pseudo-bulk analysis on the pathogenic subpopulations using the AggregateExpression function of the “Seurat” R package. Genes with a base mean > 10 and an adjusted *P* < 0.05 were identified as more robust DEGs.

### 2.6 Gene regulatory network inference via pySCENIC

To explore upstream transcriptional networks governing pathogenic cell-fate decisions, pySCENIC (v0.12.1) was used under Python (v3.7) for gene regulatory network (GRN) inference (15). Subpopulations from over 500 cells in the AD/AA dataset were downsampled to 500 and compiled into a loom object. First, GRNBoost2 inferred raw transcription factor (TF)-target co-expression modules. Second, cis-regulatory motifs within ±10 kbp of TSS were scanned via the hg38-cisTarget database to prune indirect targets, yielding high-confidence regulons. Third, AUCell scored single-cell regulon activity. Finally, the regulator-specific scores (RSSs) were calculated for pathogenic subgroups of differentially expressed comorbid genes, and rank scatterplots were plotted based on the rankings. Simultaneously, upstream core TFs associated with comorbid genes were identified via reverse-gene-based analysis, and an interaction network diagram was constructed.

### 2.7 *In silico* perturbation

To evaluate the pathogenic roles of core genes associated with comorbidity and their upstream transcription factors, Geneformer was first employed for *in silico* overexpression of the core genes, followed by the application of scTenifoldKnk to *in silico* knock out these genes. Finally, CellOracle was used to computationally perturb the upstream transcription factors.

Geneformer is a Transformer-based deep learning model (16). For network analysis, a 6-layer pre-trained Geneformer foundation transformer model (GF-6L-30M-I2048, v0.0.1) was deployed under Python (v3.10). The model was fine-tuned by retaining the pathogenic and effector cell subpopulations from the AD/AA dataset, with a linearly decaying learning rate of 5e-5, L2 regularization strength of 0.001, 500 warm-up steps, 10 training epochs, and GPU acceleration. The number of genes per cell was fixed at 2048 via adaptive truncation or padding. Then, we calculated cosine similarities between the healthy, overexpression, random, and disease states and determined the extent to which overexpression of core genes drives cells toward the disease state.

We performed tensor decomposition-based virtual knockouts of core genes using the R package scTenifoldKnk (17). After resetting gene expression signals to zero, gene networks were reconstructed using multiple cell subsets (1,000 cells per network, 10 networks in total), and the changes in the network matrices before and after this process were compared. Then, we calculated the Z-score and log_2_ Fold Change (log_2_FC) for each gene; features with log_2_FC > 4 were defined as highly disrupted, and those with 3 < log_2_FC ≤ 4 were considered moderately disrupted.

Based on the TF ranking analysis across cell subsets, pathogenic subpopulations and their neighboring healthy control states were filtered out. In Python 3.10, we used CellOracle (v0.20.0) (18) to perform virtual perturbations on TFs upstream of key genes to predict changes in cell fate trajectories and potential differentiation. First, we performed KNN gene expression imputation on the dataset with an interpolation rate of 2.5% of the total number of samples. Subsequently, the Links module was used to infer the cell subpopulation’s GRN, set the expression levels of the TFs identified by pySCENIC to 0, and refit the GRN. Finally, the migration trends of the downstream transcriptional states were simulated, generating a vector field diagram.

### 2.8 Cell-cell communication analysis

We used the R packages CellChat and multinichenetr to investigate cell-cell communication between pathogenic cell subsets and effector cells in order to elucidate the roles of key genes. To build a comprehensive database, the native CellChatDB was integrated with the CellPhoneDB (v5) database using the updateCellChatDB function. Next, the minimum cell threshold for each subpopulation was set to 5, and ligand-receptor interactions were filtered based on adjusted *P* < 0.05. The netVisual_bubble function was then used to visualize ligand-receptor pairs and generate comparative bubble plots. For multinichenetr, the expression fraction threshold (fraction_cutoff) was set to 0.03, and the minimum sample proportion was set to 0.5; ggplot2 was used to generate multi-sided directional network diagrams.

### 2.9 Spatial transcriptomic deconvolution and cellular niche identification

Spatial deconvolution was performed using cell2location (v0.1.5) (19) in Python 3.10 to map subpopulations annotated from single-cell transcriptomics to spatial transcriptomic (ST) data. First, a reference profiles matrix was derived from the scRNA-seq dataset using RegressionModel to estimate the characteristic expression of each cell type. Subsequently, the cell2location model was trained on the spatial dataset with a detection threshold of 20, a batch size of 4096, and the expected number of cells per location set to 10. The optimization was run for up to 30000 epochs, using GPU acceleration to ensure model convergence. The model can effectively estimate the probability of cell type composition at each spatial point, enabling high-resolution mapping of cell type-specific spatial distributions within a sample. We also used a Non-Negative Matrix Factorization (NMF) model to identify co-enriched cell types within the tissue space, defining biologically meaningful cell niches.

### 2.10 Xenium spatial mapping and interaction network analysis

Absolute physical micron coordinates of the Xenium dataset were converted into relative pixel coordinates corresponding to the local coordinate system of the raw tissue images. The annotated cell subpopulations were mapped as ultra-high-resolution fluorescence-like scatter points and overlaid on a dark-field DAPI background. The regional microenvironment enriched with pathogenic subpopulations exhibiting high expression of core comorbid genes, along with their adjacent effector cells, was extracted after clipping. Concurrently, we used Squidpy to construct an average intercellular interaction matrix, which was converted into a weighted adjacency network using igraph to visualize the colocalization and interaction patterns of cell clusters within the spatial organization.

### 2.11 Cell culture and quantitative real-time PCR

The human keratinocyte cell line HaCaT (Cat. C5070) and the human bronchial epithelial cell line BEAS-2B (Cat. C5382) were purchased from Shanghai Biocode Biotech Co., Ltd. (Shanghai, China) and authenticated by short tandem repeat profiling. The SV40T-immortalized human dermal fibroblast (HDF) cell line (Cat. CP-H103Y) was purchased from Procell Life Science & Technology Co., Ltd. (Wuhan, China) and authenticated by Vimentin immunofluorescence staining. All cell lines were cultured in Dulbecco’s Modified Eagle Medium (DMEM) supplemented with 10% fetal bovine serum and 1% penicillin-streptomycin (100 U/mL), and routinely maintained in a humidified incubator at 37 °C with 5% CO_2_.

HaCaT, BEAS-2B, and HDF cells were seeded into 12-well plates at a density of 1.5 × 10^5^ cells/mL (1 mL per well). The experiments were divided into a blank control group (Control) and a cytokine treatment group (IL-4 + IL-13). After 24 h of incubation to allow cell attachment and confluence, the serum-containing medium was discarded. After washing with PBS, the medium was replaced with serum-free DMEM for 2 h of starvation. The treatment group was stimulated with a combination of recombinant human IL-4 (20 ng/mL; Cat. CX03, Novoprotein, China) and recombinant human IL-13 (20 ng/mL; Cat. CC89, Novoprotein, China) for 24 h (20–23), while the control group received an equal volume of serum-free medium and was cultured for the same duration before cell harvesting. Four independent biological replicates were performed for each group.

Total cellular RNA was extracted using the Trizol method, and the concentration and purity (A260/A280 ratio) of the RNA were determined using a spectrophotometer (NanoDrop). One microgram of total RNA was reverse-transcribed to synthesize cDNA according to the manufacturer’s instructions for the reverse transcription kit (Cat. RR047A, Takara, China). The TB Green Premix Ex Taq II kit (Cat. RR820A, Takara, China) was used to quantify the expression levels of comorbid genes by real-time PCR, with GAPDH as the endogenous control, on a StepOne Plus PCR system (Thermo Fisher, United States). Each biological sample was analyzed in technical triplicate. The relative expression levels of the target genes were calculated using the 2^-ΔΔCT^ method. Statistical analysis was performed in R using the unpaired Student’s t-test, and *P* < 0.05 was considered statistically significant. The primer sequences are detailed in the **Supplementary Materials** (**Supplementary Table 2**).

## 3 Results

### 3.1 Batch correction and differential expression analysis

**Figure 2:**
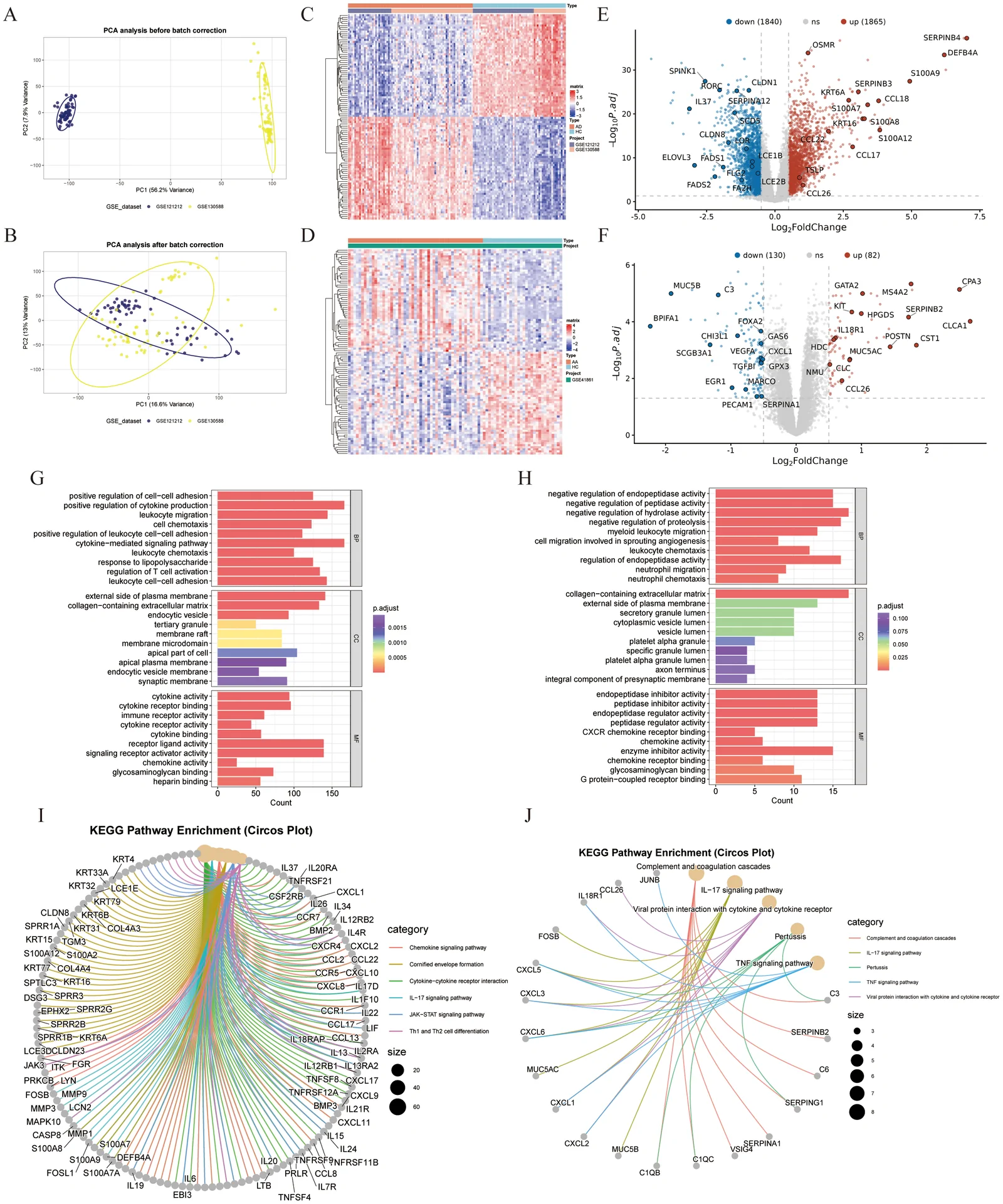
Batch-effect correction, differentially expressed genes, and functional enrichment analyses of the AD and AA datasets. **(A, B)** PCA plots of the AD dataset before **(A)** and after **(B)** batch-effect correction. **(C, D)** Hierarchical clustering heatmaps of the DEGs in AD **(C)** and AA **(D)**. **(E, F)** Volcano plots of the DEGs in AD **(E)** and AA **(F)**, with representative genes annotated in the plots. **(G, H)** GO functional enrichment bar plots for DEGs in AD **(G)** and AA **(H)**. **(I, J)** KEGG pathway enrichment Circos plots for DEGs in AD **(I)** and AA **(J)**. AD, atopic dermatitis; AA, allergic asthma; PCA, principal component analysis; DEGs, differentially expressed genes; GO, Gene Ontology; KEGG, Kyoto Encyclopedia of Genes and Genomes.

As shown in **Figures 2A, B**, a noticeable batch effect was observed after merging the AD datasets GSE121212 and GSE130588. After removing the batch effect using ComBat, the variance explained by the first principal component decreased from 56.2% to 16.6%, indicating a significant reduction in the batch effect, and the expression levels across all samples became consistent. After quality control, differential expression analysis was performed on the AD and AA datasets using the limma package. In the AD dataset, 3705 DEGs were identified (1865 up-regulated genes and 1840 down-regulated genes), and in the AA dataset, 212 DEGs were identified (82 up-regulated genes and 130 down-regulated genes). The heatmaps and volcano plots of the DEGs are shown in **Figures 2C–F**.

Functional enrichment analysis revealed both shared and distinct immunopathological signatures between AD and AA (**Figures 2G–J**). In AD, biological processes and pathways were characterized by extensive leukocyte migration, broad cytokine-mediated signaling, and receptor-ligand interactions (e.g., Cytokine-cytokine receptor interaction, JAK-STAT signaling, and Chemokine signaling pathway), alongside a mixed adaptive immune response involving Th1 and Th2 cell differentiation and the IL-17 signaling pathway, accompanied by alterations in epidermal structural integrity (Cornified envelope formation). In contrast, AA was characterized by proteolytic imbalance and alterations in secretory structures, with activation of the IL-17 and TNF pathways and overactivation of the complement and coagulation cascades, suggesting a distinct innate immune mechanism.

### 3.2 WGCNA analysis and identification of comorbid genes

**Figure 3:**
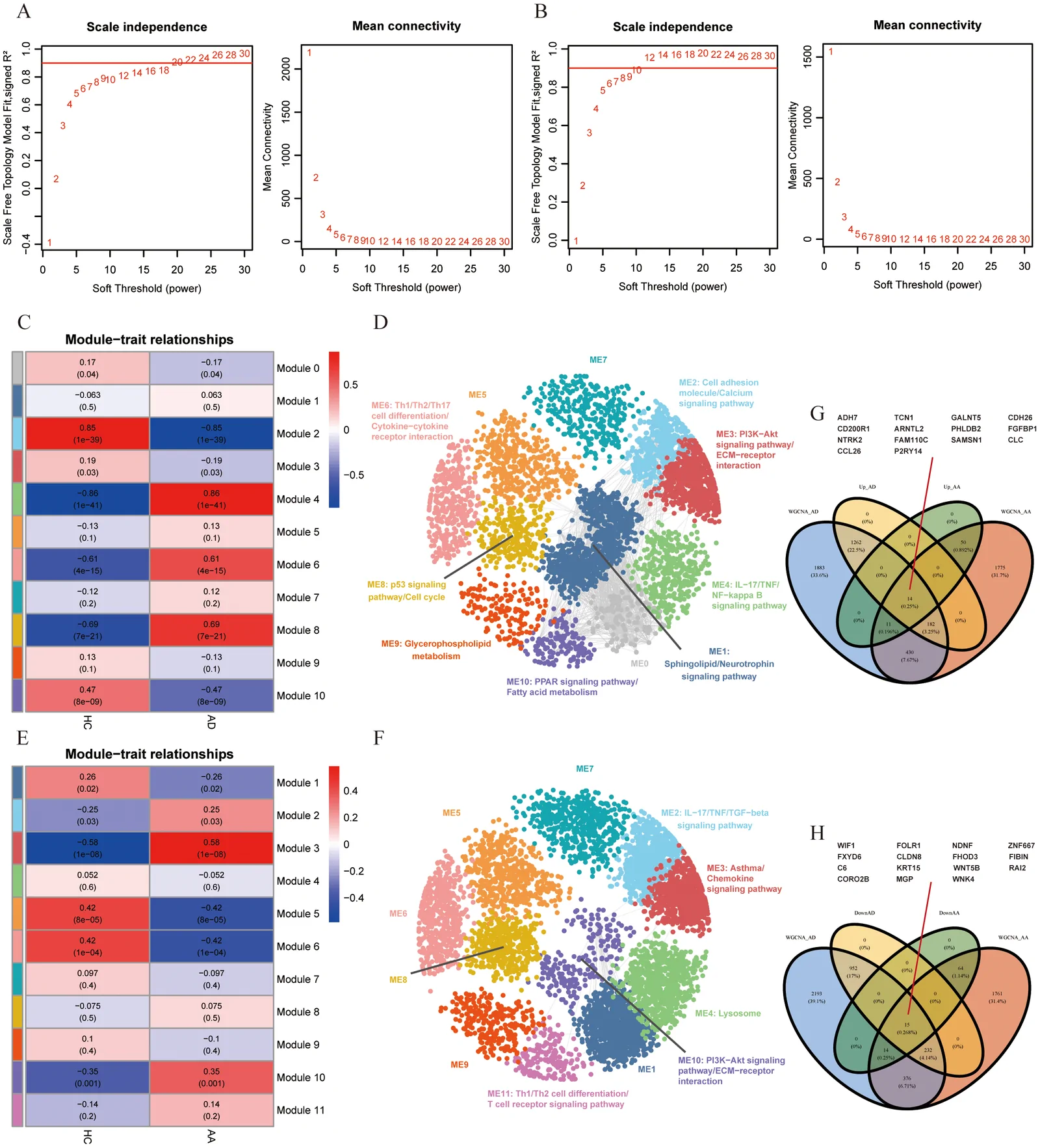
WGCNA co-expression network and intersection analysis of the AD and AA datasets. **(A, B)** Soft-thresholding power selection plots for the AD **(A)** and AA **(B)** datasets, with the left panel showing the scale-free topology model fit index (R^2^) and the right panel showing the mean connectivity. **(C, D)** Module-trait relationship heatmaps for the AD **(C)** and AA **(D)** datasets. **(E, F)** Co-expression network module diagrams for the AD **(E)** and AA **(F)** datasets, and annotated with pathways significantly enriched in the module. **(G, H)** Venn diagrams intersecting DEGs and genes from WGCNA trait-related modules in AD and AA, showing the intersection of up-regulated genes **(G)** and down-regulated genes **(H)** between the two conditions. AD, atopic dermatitis; AA, allergic asthma; WGCNA, weighted gene co-expression network analysis; DEGs, differentially expressed genes.

WGCNA was performed to identify gene modules significantly positively and negatively correlated with the clinical traits of the AD and AA datasets. According to **Figures 3A, B**, the soft-thresholding powers (β) for the AD and AA datasets were set to 20 and 10, respectively, ensuring that the co-expression networks conformed to the scale-free distribution topology. In the AD dataset, 10 gene modules (excluding module 0) were identified, as shown in **Figure 3C**. Modules 4 (*r* = 0.86, *P* = 1×10^-41^), 6 (*r* = 0.61, *P* = 4×10^-15^), and 8 (*r* = 0.69, *P* = 7×10^-21^) were selected as significantly positively correlated modules, while module 2 was selected as the significantly negatively correlated module (*r* = -0.85, *P* = 1×10^-39^). These modules were enriched in pathways associated with AD pathogenesis, including Th1/Th2/Th17 signaling (**Figure 3E**). In the AA dataset, a total of 11 gene modules were obtained, as shown in **Figure 3D**. Module 3 (*r* = 0.58, *P* = 1×10^-8^) was selected as the significantly positively correlated module. Since no module exhibited a correlation coefficient of less than -0.5, modules 5 (*r* = -0.42, *P* = 8×10^-5^) and 6 (*r* = -0.42, *P* = 1×10^-4^) were chosen as the significantly negatively correlated modules. Among them, module 3 was enriched in asthma and chemokine pathways (**Figure 3F**). Finally, through Venn diagram analysis intersecting the DEGs and the respective trait-related modules, 14 co-upregulated genes and 15 co-downregulated genes were identified (**Figures 3G, H**), which were utilized for subsequent feature engineering and diagnostic model construction.

### 3.3 Machine learning-based model construction

**Figure 4:**
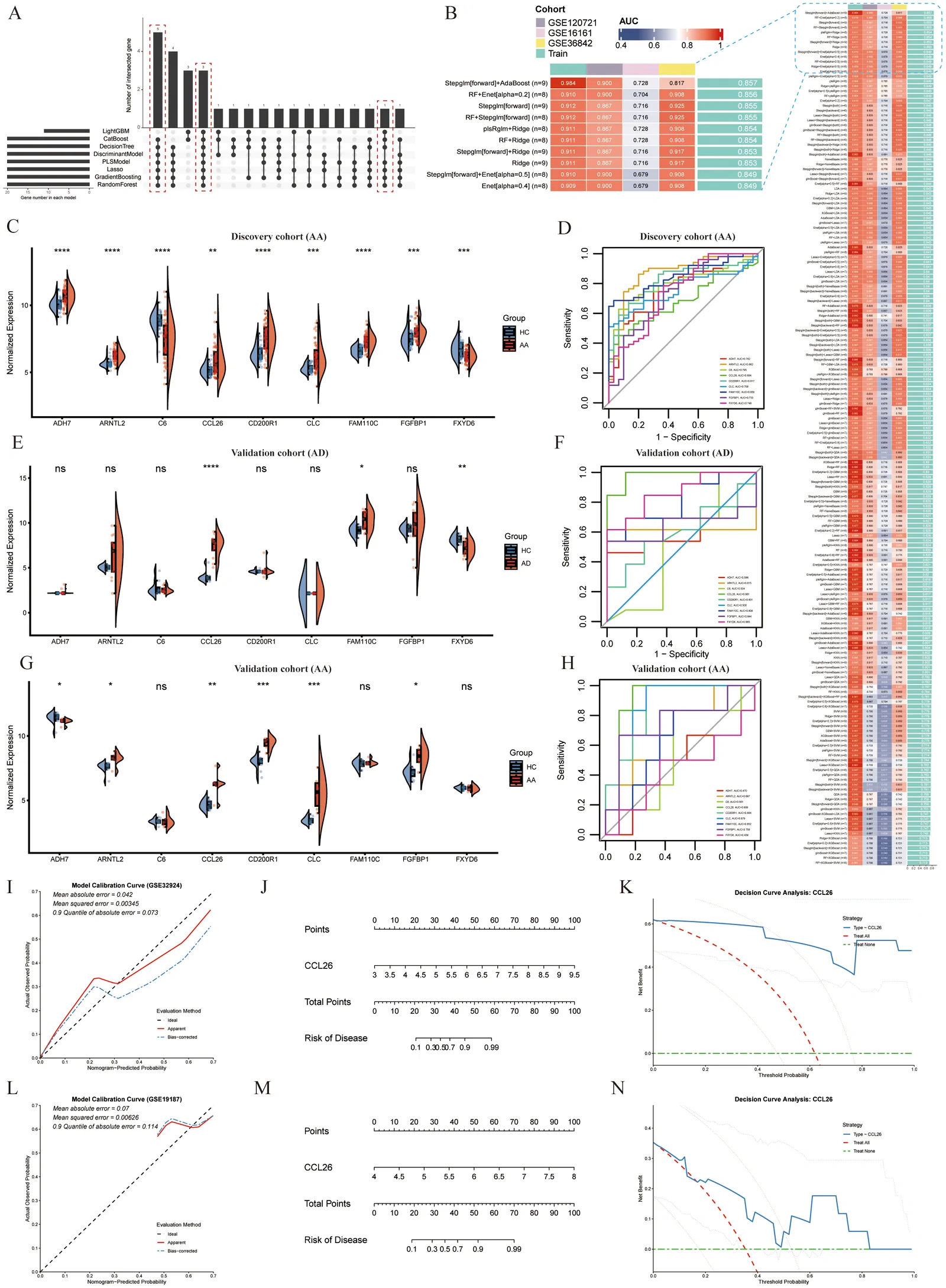
Machine learning feature selection, diagnostic model construction, and validation of CCL26. **(A)** UpSet plot of eight feature selection algorithms screening for comorbid genes, with the red dashed box highlighting genes selected by seven or more algorithms. **(B)** AUC heatmap of 175 machine learning model combinations across the training set and testing sets (GSE120721, GSE16161, and GSE36842), with an enlarged view showing the top 10 models based on average AUC. **(C, D)** Differential expression and ROC curves of nine candidate genes in the AA discovery cohort (GSE41861). **(E, F)** Differential expression and ROC curves of nine candidate genes in the AD validation cohort (GSE32924). **(G, H)** Differential expression and ROC curves of nine candidate genes in the AA validation cohort (GSE19187). **(I, L)** Calibration curves of the nomogram models in the GSE32924 and GSE19187 cohorts. **(J, M)** CCL26-based nomograms in the GSE32924 and GSE19187 cohorts. **(K, N)** DCA curves of the CCL26-based nomograms in the GSE32924 and GSE19187 cohorts. AUC, area under the receiver operating characteristic curve; ROC, receiver operating characteristic; AA, allergic asthma; AD, atopic dermatitis; mRNA, messenger RNA; DCA, Decision curve analysis. *P < 0.05, **P < 0.01, ***P < 0.001, ****P < 0.0001; ns, no statistical significance.

First, a feature subset was extracted from the AD gene set by filtering for 29 comorbid genes. The samples were then split into training and test sets in a 7:3 ratio. During training, 5-fold repeated cross-validation was used to obtain optimal model parameters and ensure stability. To capture non-linear relationships and interactions among genes across different dimensions, the eight robust feature selection algorithms described in the methods section were used to select features for the comorbid genes. The results of feature selection are shown in **Figure 4A**. Genes appearing in at least 7 algorithms were selected for subsequent diagnostic model construction (up-regulated genes: CLC, CCL26, ADH7, FAM110C, FGFBP1, CD200R1, and ARNTL2; down-regulated genes: FXYD6 and C6).

Using systematic permutations and combinations of 15 machine learning models and the enumeration method, the merged AD dataset from GSE121212 and GSE130588 was trained. Models utilizing more than 5 genes were retained, ultimately resulting in 175 valid models. The AUC values for the training and testing sets, along with their average AUCs, were calculated, as shown in **Figure 4B**. The models with superior average AUC values included Stepglm[forward]+AdaBoost, RF+Enet[alpha=0.2], Stepglm[forward], RF+Stepglm[forward], and plsRglm+Ridge. Among them, the Stepglm[forward]+AdaBoost model achieved an AUC of 0.984 on the training set, and AUCs of 0.900 for GSE120721, 0.728 for GSE16161, and 0.817 for GSE36842 on the testing sets, yielding the highest average AUC among all evaluated models. Through the AA discovery cohort (GSE41861), it was confirmed that the model composed of these 9 genes (CLC, CCL26, ADH7, FAM110C, FGFBP1, CD200R1, ARNTL2, FXYD6, and C6) also demonstrated strong discriminative ability for diagnosing AA (**Figures 4C, D**).

However, in the validation cohorts of AD (GSE32924) and AA (GSE19187), only the gene expression level and significance of CCL26 remained consistent, and it exhibited favorable diagnostic performance (GSE32924: AUC = 0.981; GSE19187: AUC = 0.909), as shown in **Figures 4E–H**. We validated the gene expression levels across all cohorts, and CCL26 was significantly up-regulated in all of them (**Supplementary Figures 1A–D**). Based on this, CCL26 was selected as the core comorbidity gene. The nomogram models constructed using the validation cohorts both demonstrated strong diagnostic efficacy, indicating that as CCL26 expression increased, the probability of developing AD (**Figures 4I–K**) and AA (**Figures 4L–N**) increased. Furthermore, the models showed good calibration and low absolute errors (GSE32924: Mean absolute error = 0.042, 0.9 Quantile of absolute error = 0.073; GSE19187: Mean absolute error = 0.07, 0.9 Quantile of absolute error = 0.114).

### 3.4 Single-cell landscape and differential expression analysis of CCL26 in AD and AA

**Figure 5:**
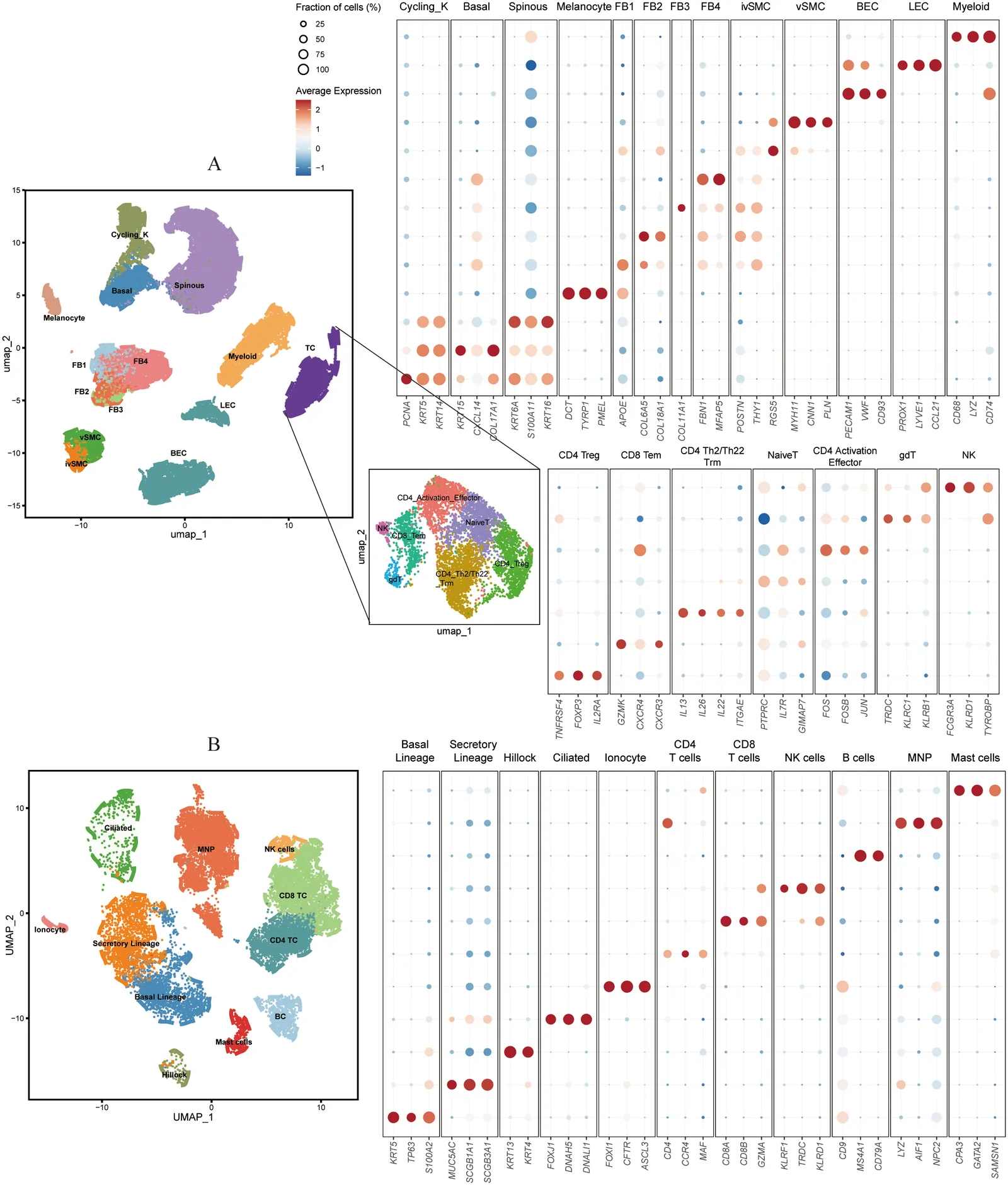
Cell type annotation in single-cell transcriptomic analysis of the AD and AA datasets. **(A)** Single-cell UMAP clustering plot of the AD dataset, identifying 13 cell types, with a dot plot showing the expression of marker genes for each cell type; the inset presents the UMAP clustering plot of T cell subsets (further classified into 7 functional subclusters), with a dot plot showing the expression of marker genes for each T cell subset. **(B)** Single-cell UMAP clustering plot of the AA dataset, identifying 11 cell types, with a dot plot showing the expression of marker genes for each cell type. AD, atopic dermatitis; AA, allergic asthma; UMAP, uniform manifold approximation and projection.

To analyze the role of the core shared gene CCL26 in AD and AA, we performed a comprehensive single-cell sequencing analysis using the single-cell datasets GSE222840 (AD) and GSE193816 (AA). After quality control, including the removal of doublets, batch effects, and ambient RNA contamination, a total of 50994 cells and 23097 genes were retained for the AD dataset, and 17233 cells and 28043 genes were retained for the AA dataset (**Supplementary Figures 2A, B**). Based on canonical marker genes, cells in the AD dataset were annotated into 13 distinct cell types (**Figure 5A**), and T cells were further classified into 7 functional subpopulations. Notably, a pathogenic subset of cutaneous effector T cells displaying a tissue-resident memory T (T_rm_) profile with combined Type 2 and Type 22 cytokine signatures (Th2/Th22 T_rm_) was identified by the co-expression of effector cytokines (IL13, IL22, IL26) and the tissue-residency marker ITGAE (24). Following the literature by Helen He et al. (25), fibroblasts were partitioned into 4 distinct subclusters: APOE+, COL6A5+COL18A1+, COL11A1+, and FBN1+MFAP5+ fibroblasts, representing lipid metabolism, an inflammatory drive, tissue repair/fibrosis, and maintenance of skin structure, respectively. Within vascular smooth muscle cells (vSMCs), we identified a population of inflammatory vascular smooth muscle cells (ivSMCs) that expressed fibroblast-like genes (POSTN, COL18A1) and chemokines (CCL26, CCL19). Gene Set Enrichment Analysis (GSEA) in **Supplementary Figure 2C** showed that inflammatory pathways, including INTERFERON_GAMMA_RESPONSE, INTERFERON_ALPHA_RESPONSE, IL6_JAK_STAT3_SIGNALING, and INFLAMMATORY_RESPONSE, were highly enriched in this subpopulation. The AA dataset was classified into 11 cell types based on representative marker genes, as shown in **Figure 5B**, and the further subpopulation classification of T cells is presented in **Supplementary Figure 2D**.

**Figure 6:**
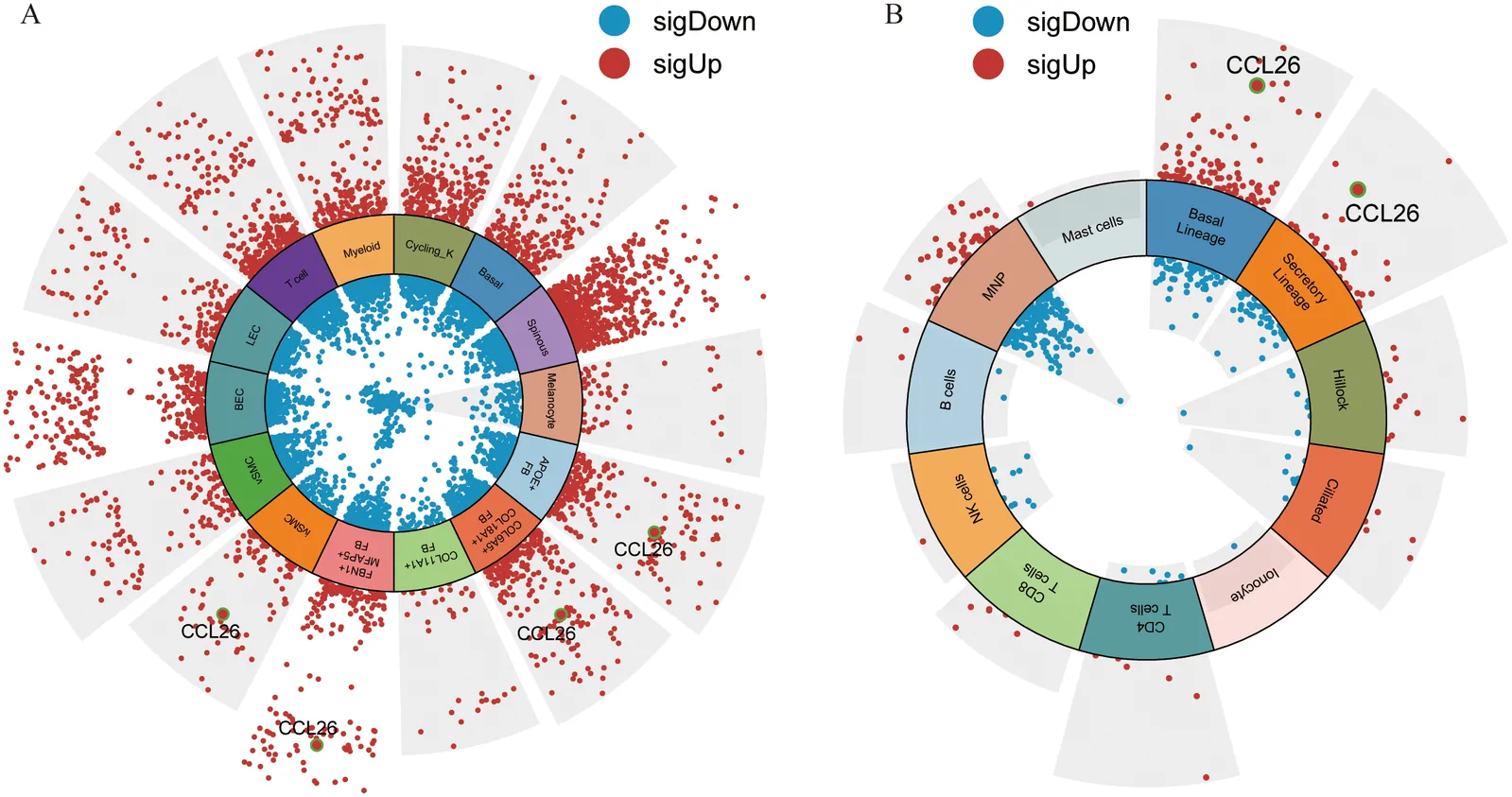
Circular scatter plots of DEGs across cell subclusters and the expression distribution of CCL26 in AD and AA. **(A, B)** Circular scatter plots of DEGs across each cell type in the AD **(A)** and AA **(B)** datasets, where the radial distance of the dots represents the degree of differential expression; the green circles in the plots highlight the position of the comorbid gene CCL26 within the corresponding cell types. AD, atopic dermatitis; AA, allergic asthma; DEGs, differentially expressed genes.

After identifying the cell types, we performed pseudo-bulk analysis to examine the differential distribution of the core share gene CCL26 across them, as shown in **Figures 6A, B**. In AD patients, CCL26 expression was markedly elevated compared to healthy controls in APOE+ FBs (log_2_FC = 6.8), COL6A5+COL18A1+ FBs (log_2_FC = 5.5), FBN1+MFAP5+ FBs (log_2_FC = 8.0), and ivSMCs (log_2_FC = 5.6). In AA patients, CCL26 expression was significantly increased compared with healthy controls in the Basal Lineage (log_2_FC = 5.8) and the Secretory Lineage (log_2_FC = 3.4).

### 3.5 Identification of disease-associated single-cell subpopulations

**Figure 7:**
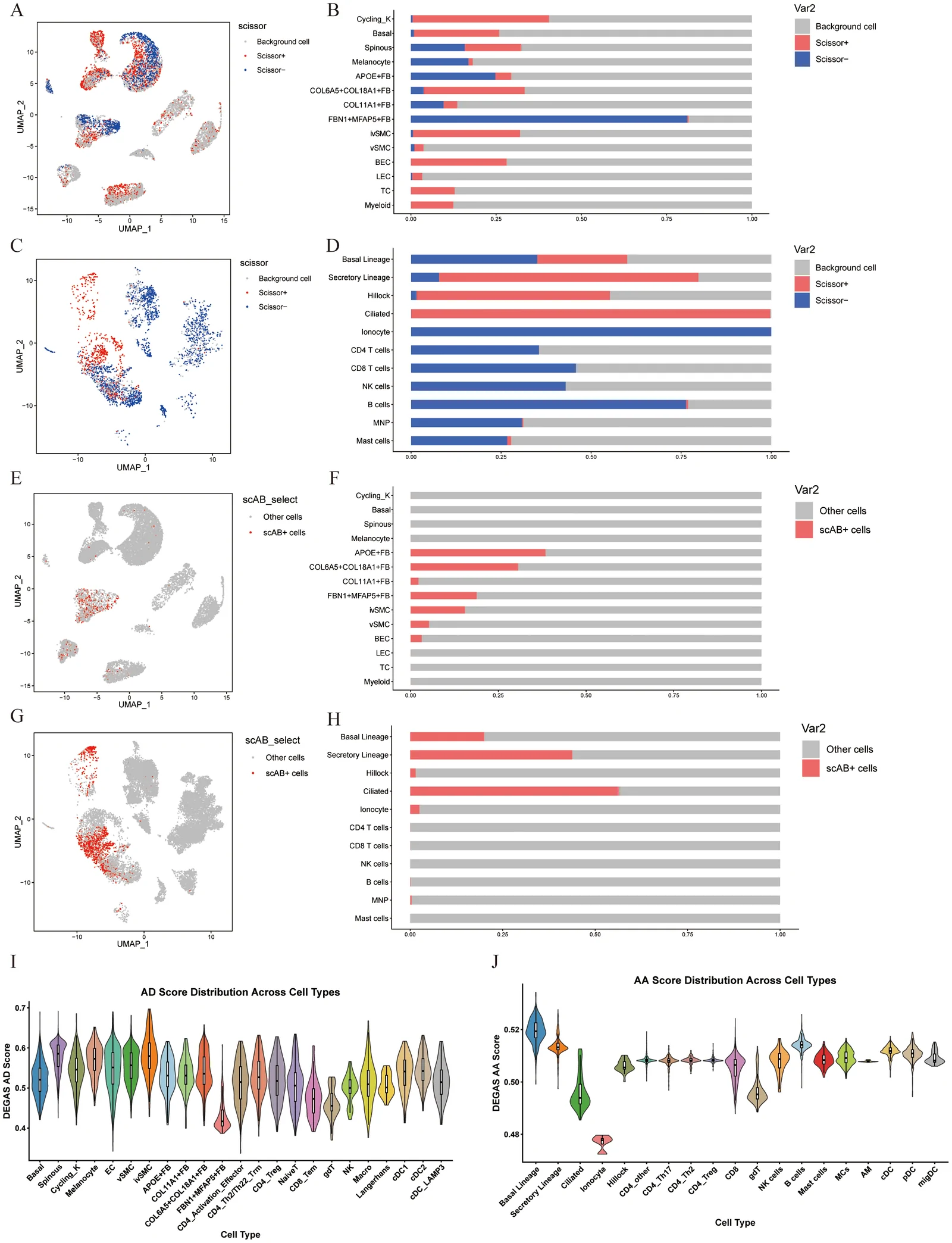
Identification of AD- and AA-associated single-cell subclusters using Scissor and scAB algorithms alongside DEGAS disease score analysis. **(A, B)** UMAP plot and bar plot after mapping the AD bulk transcriptomic data (GSE121212/GSE130588) to the single-cell dataset using the Scissor algorithm, where red (Scissor+) represents the high-risk cell subcluster, blue (Scissor-) represents the low-risk cell subcluster, and gray (Background cell) represents background cells. **(C, D)** UMAP plot and bar plot after mapping the AA bulk transcriptomic data (GSE41861) to the single-cell dataset using the Scissor algorithm. **(E, F)** UMAP plot and bar plot of AD disease-associated cell subclusters identified by the scAB algorithm, where red (scAB+ cells) represents disease-associated positive cells and gray (Other cells) represents other cells. **(G, H)** UMAP plot and bar plot of AA disease-associated cell subclusters identified by the scAB algorithm. **(I, J)** Violin plots of disease scores across each cell subcluster in AD and AA, which were calculated based on the DEGAS algorithm. AD, atopic dermatitis; AA, allergic asthma; UMAP, uniform manifold approximation and projection.

The Scissor algorithm was used to map bulk transcriptomic data from AD and AA back to single-cell datasets, identifying disease-associated single-cell subpopulations. **Figures 7A–D** show the high-risk (Scissor+) and low-risk (Scissor-) cell subpopulations associated with AD and AA pathogenesis. In the AD dataset, the high-risk subpopulations were mainly found in keratinocytes, ivSMCs, blood vascular endothelial cell (BECs), and COL6A5+COL18A1+ FBs, whereas in the AA dataset, they were found in airway epithelial cells. To further validate the stability of these high-risk subpopulations and the role of the shared gene CCL26, scAB analysis was performed (**Figures 7E–H**). The primary pathogenic subpopulations in AD were annotated as APOE+ FBs, COL6A5+COL18A1+ FBs, FBN1+MFAP5+ FBs, and ivSMCs, while those in AA were secretory lineage, basal lineage, and ciliated cells. The FindMarkers function was used to analyze differential expression between high-risk cells (scAB+) and other cells within these subpopulations. The results revealed that CCL26 was significantly expressed in the pathogenic cells of ivSMCs in AD (log_2_FC = 0.68), as well as secretory lineage (log_2_FC = 0.76) and basal lineage (log_2_FC = 1.49) cells in AA (**Supplementary Figures 3A–I**). Finally, a deep learning model with a three-layer network architecture was constructed on the AD and AA single-cell datasets using the runCCMTLBag function from the DEGAS package, followed by 5 Bootstrap resampling iterations to project bulk disease status into single-cell scores and calculate the disease probability for each subpopulation in AD and AA, as shown in **Figures 7I, J**. Among the AD cell subpopulations, ivSMCs exhibited the highest disease score, whereas basal lineage cells showed the highest score among the AA cell subpopulations.

### 3.6 Verification of CCL26 perturbation effects via Geneformer and scTenifoldKnk

**Figure 8:**
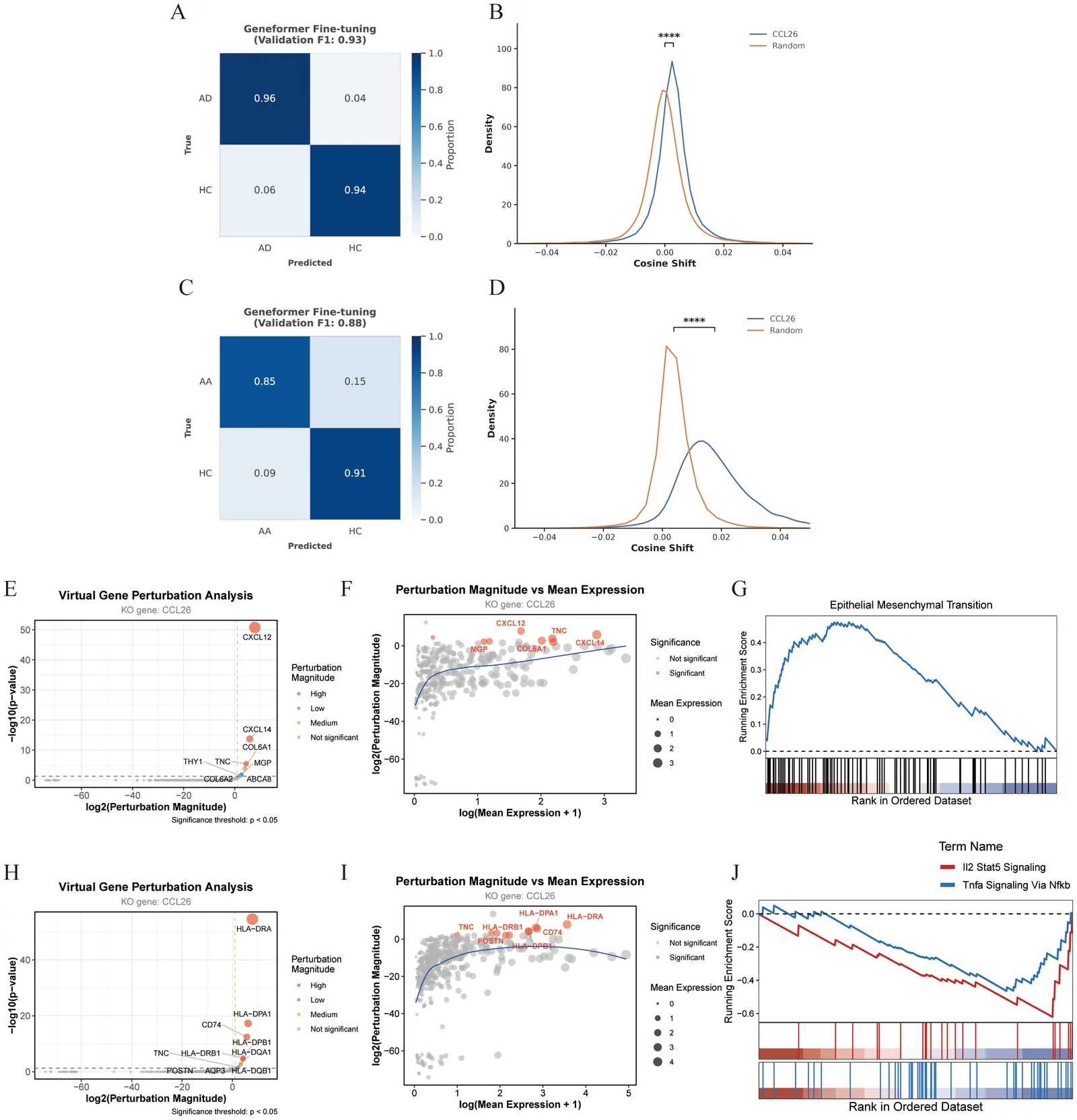
Validation of CCL26 perturbation effects using Geneformer and scTenifoldKnk. **(A, C)** Confusion matrices of the fine-tuned Geneformer models for the AD and AA datasets. **(B, D)** Density plots of the cosine shift driving cell state transitions induced by virtual overexpression of CCL26 compared with random control genes in the AD and AA datasets. **(E, F)** Volcano plot and scatter plot showing the relationship between perturbation strength and mean gene expression for the virtual knockout of CCL26 based on scTenifoldKnk in the AD dataset, with the blue curve representing the fitted trendline. **(G)** GSEA score curves following virtual knockout of CCL26 in the AD dataset. **(H, I)** Volcano plot and scatter plot showing the relationship between perturbation strength and mean gene expression for the virtual knockout of CCL26 based on scTenifoldKnk in the AA dataset. **(J)** GSEA score curves following virtual knockout of CCL26 in the AA dataset. (****P < 0.0001). AD, atopic dermatitis; AA, allergic asthma; GSEA, gene set enrichment analysis.

To systematically demonstrate the significance of the core shared gene CCL26 in the pathogenesis of AD and AA, we performed *in silico* virtual overexpression of CCL26 in healthy control states via Geneformer and *in silico* virtual knockout of CCL26 in disease states via scTenifoldKnk to observe the regulatory effects of CCL26 perturbations. The Geneformer foundation model (GF-6L-30M-I2048) was fine-tuned to the distinct cell-type compositions of AD and AA, and its predictive performance was evaluated on the respective validation sets using normalized confusion matrices and F1-Scores. **Figures 8A, C** demonstrated that, after fine-tuning, the models exhibited robust discrimination between disease states and healthy controls (F1-Scores: AD = 0.93; AA = 0.88). By comparing the overexpression of random control genes and CCL26, we found that virtual overexpression of CCL26 induced a transition of cells from a healthy state to a disease-like phenotype (**Figures 8B, D**).

Subsequently, we performed a network-based virtual knockout of CCL26 using scTenifoldKnk. In the AD dataset, virtual CCL26 ablation perturbed key genes, including CXCL12, CXCL14, COL6A1, and TNC (**Figures 8E, F**), and this perturbation was accompanied by a massive re-organization of the epithelial-mesenchymal transition (EMT) pathway (**Figure 8G**). Concurrently, in the AA dataset, virtual CCL26 ablation perturbed human leukocyte antigen (HLA) class II molecules, alongside CD74, TNC, and POSTN, as the most significantly perturbed genes in the network (**Figures 8H, I**), and this was followed by deep disruption of the topological homeostatic networks of the IL2_STAT5 and TNFA_SIGNALING_VIA_NFKB pathways (**Figure 8J**).

### 3.7 Upstream transcriptional regulation and cell-state control of CCL26

**Figure 9:**
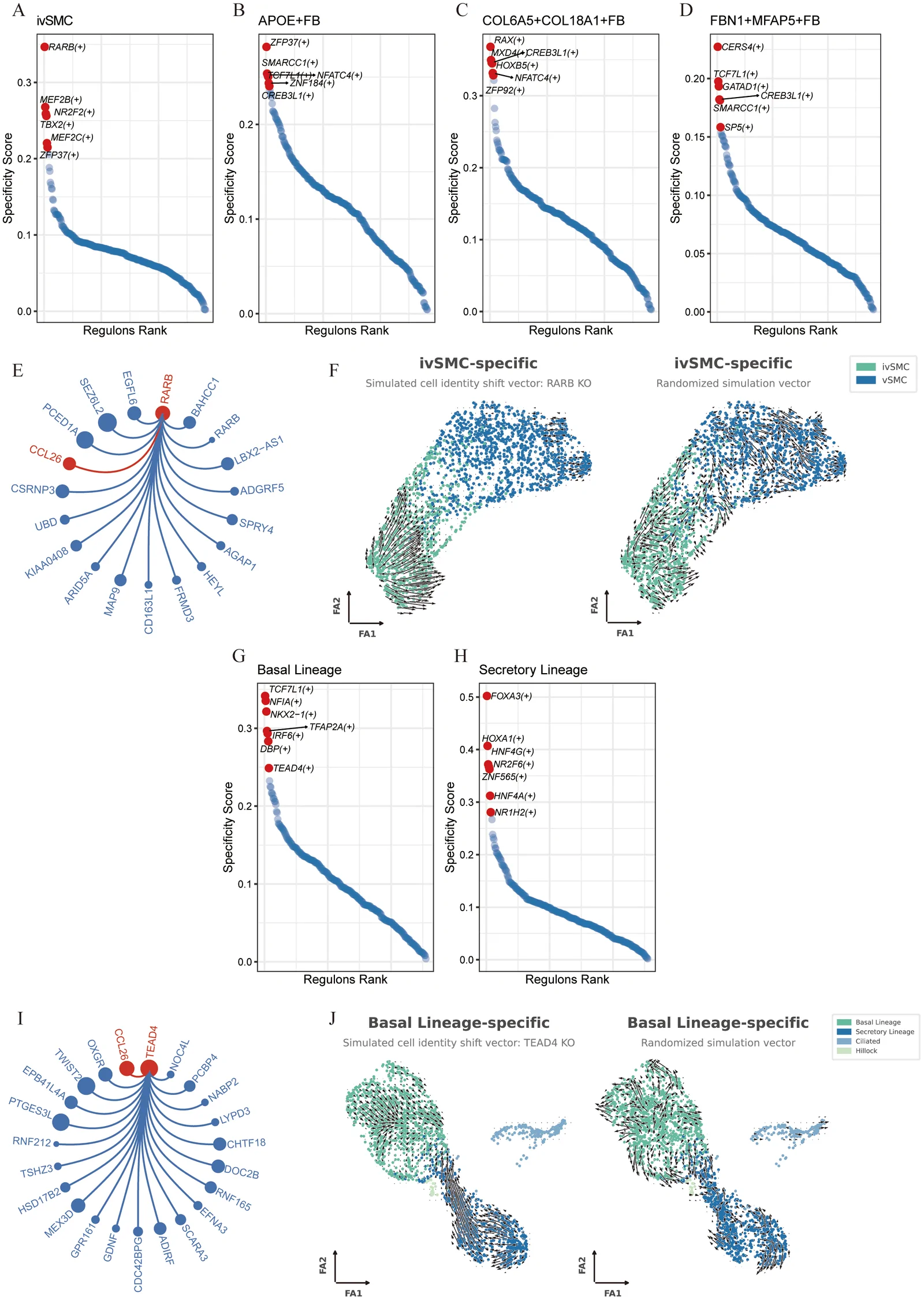
Upstream transcriptional regulation of CCL26 and its impact on the fate of cell subclusters. **(A–D)** RSS ranking plots for the ivSMC, APOE+FB, COL6A5+COL18A1+FB, and FBN1+MFAP5+FB subclusters. **(E)** Transcription factor regulatory network of RARB in ivSMC, with RARB and its target gene CCL26 shown in red, and other target genes in blue. **(F)** Vector fields and trajectory changes of cell state transitions in ivSMC simulated by CellOracle following RARB knockout (left) and random knockout (right). **(G, H)** RSS ranking plots for the Basal Lineage and Secretory Lineage subclusters. **(I)** Transcription factor regulatory network of TEAD4 in the Basal Lineage, with TEAD4 and its target gene CCL26 shown in red, and other target genes in blue. **(J)** Vector fields and trajectory changes of cell state transitions in the Basal Lineage simulated by CellOracle following TEAD4 knockout (left) and random knockout (right). RSS, regulon specificity score; ivSMC, inflammatory vascular smooth muscle cell; FB, fibroblast.

In conjunction with pseudo-bulk differential expression analysis, TF activity inference was performed on the clinical pathogenic high-risk subpopulations identified by Scissor and scAB to elucidate the upstream core molecular mechanisms driving the aberrant activation of CCL26. RSS rank plots showed abnormally high activity for multiple TF regulons in pathogenic subpopulations of both AD (**Figures 9A–D**) and AA (**Figures 9G, H**). A CCL26 regulatory network was subsequently constructed through co-variation trend analysis of CCL26 with its upstream transcription factors and enrichment analysis of binding motifs within the promoter region. RARB (**Figure 9E**) and TEAD4 (**Figure 9I**) were identified as upstream transcriptional regulators of CCL26 in AD and AA, respectively, with RARB highly expressed in ivSMCs and TEAD4 in the basal lineage. To further investigate whether the specific transcriptional regulators RARB and TEAD4 influenced the cell states of these pathogenic subpopulations, *in silico* knockouts were performed using CellOracle. After setting the expression of RARB and TEAD4 to zero, respectively, a regular perturbation vector field oriented toward vSMCs was observed in the ivSMC subpopulation of AD (**Figure 9F**). In AA, the vector field was regularly oriented toward an intermediate state between the basal and secretory lineages (**Figure 9J**). In contrast, random knockouts of transcription factors failed to produce orderly, regular vector fields.

### 3.8 Intercellular CCL26 signaling of pathogenic subpopulations

**Figure 10:**
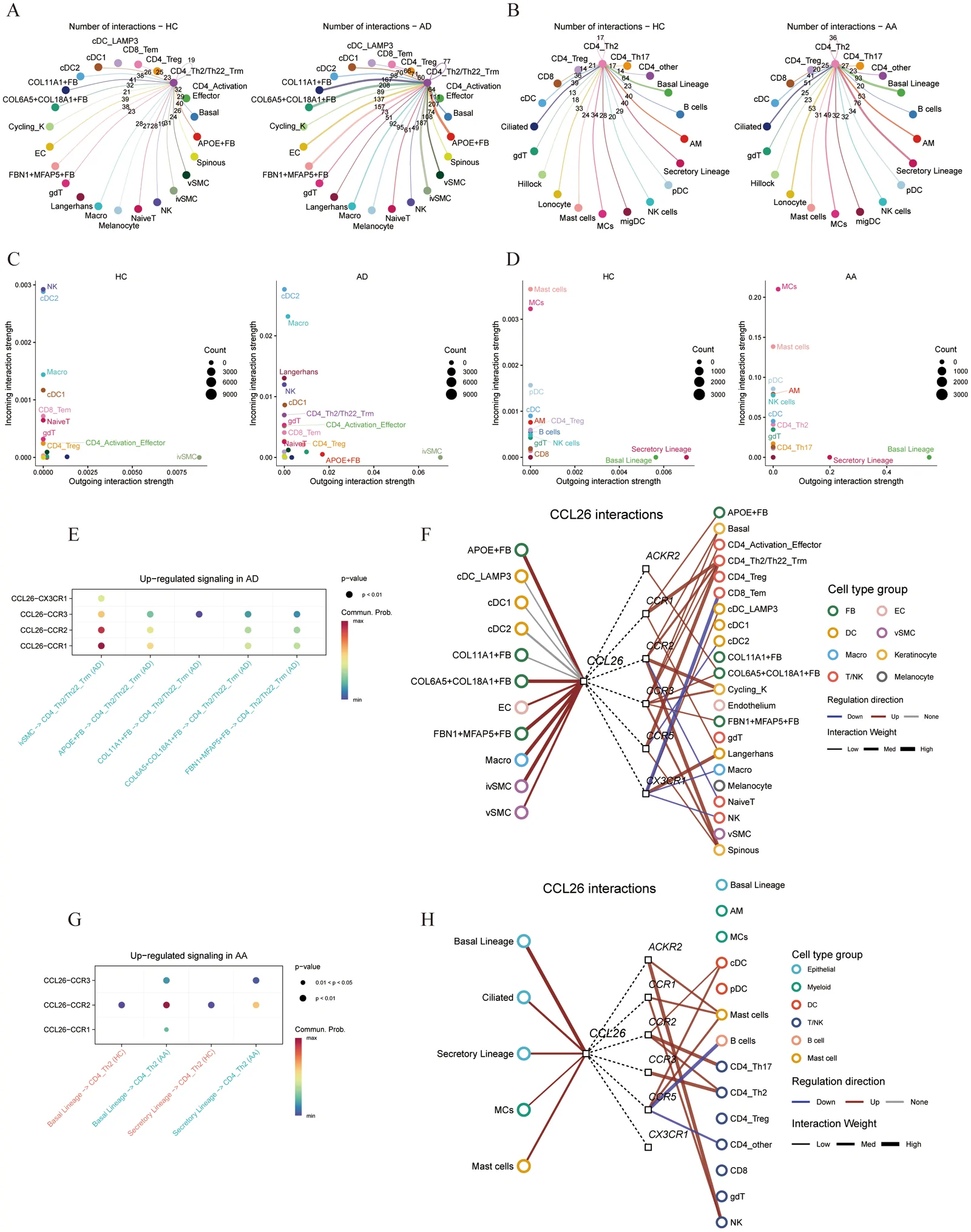
CellChat and multinichenetr cell-cell communication analyses reveal the role of CCL26 signaling in AD and AA. **(A, B)** Interaction network plots between each cell subcluster and Th2/Th22 T_rm_ cells (AD) or Th2 cells (AA) in healthy controls and disease groups across the AD and AA datasets. **(C, D)** Scatter plots of incoming and outgoing interaction strengths for CCL26 ligand-receptor signaling across each cell type in healthy controls and disease groups within the AD and AA datasets. **(E, G)** Bubble plots of CCL26-related ligand-receptor communication between disease-associated subclusters and Th2/Th22 T_rm_ cells (AD) or Th2 cells (AA) in the AD and AA datasets. **(F, H)** Intercellular communication network plots mediated by CCL26 and its receptors (ACKR2, CCR1-3, CCR5, CX3CR1) in AD and AA. AD, atopic dermatitis; AA, allergic asthma; T_rm_, tissue-resident memory T cells.

We analyzed intercellular interactions between various cell types and effector T-cell subsets and found that communication intensity between ivSMCs, APOE+ FBs, COL11A1+ FBs, and COL6A5+COL18A1+ FBs and Th2/Th22 T_rm_ cells was markedly enhanced in AD (**Figure 10A**). In AA, the communication intensities of the basal lineage and secretory lineage with Th2 cells were also strengthened to a certain extent (**Figure 10B**). To clarify the specificity of the CCL26 communication response, we mapped global cellular interactions using CellChat signaling pathway analysis. As illustrated in **Figures 10C, D**, compared with healthy controls, incoming CCL26 signaling targeting Th2/Th22 T_rm_ in AD and Th2 cells in AA was substantially elevated. In healthy controls and AD tissues, ivSMCs consistently acted as the primary CCL26 signaling donors, and this output was further amplified in AD. In AA, however, the basal lineage showed strongly outgoing CCL26 signaling. The ligand-receptor pair analyses in **Figures 10E, G** further substantiated these findings; in AD, multiple CCL26-related ligand-receptor communication signals linking ivSMCs with Th2/Th22 T_rm_ cells were significantly elevated, whereas in AA, the CCL26-associated cellular communication intensity related to the basal lineage was higher, echoing the pySCENIC findings from the previous section. Furthermore, in **Figures 10F, H**, we employed multinichenetr to compare differential cell-cell communication between the disease groups and healthy controls, revealing that cellular communication via CCL26-related chemokine receptors between the critical pathogenic cell subpopulations and effector T-cell subsets in AD and AA was markedly stronger than in healthy controls (in AD, the signaling of CCL26 secreted by pathogenic subpopulations towards CCR1, CCR2, CCR3, and CCR5 in Th2/Th22 T_rm_ cells was enhanced; in AA, the signaling of CCL26 secreted by pathogenic subpopulations towards CCR2 and CCR3 in Th2 cells was enhanced).

### 3.9 Spatial niche of pathogenic subpopulations and effector cells

**Figure 11:**
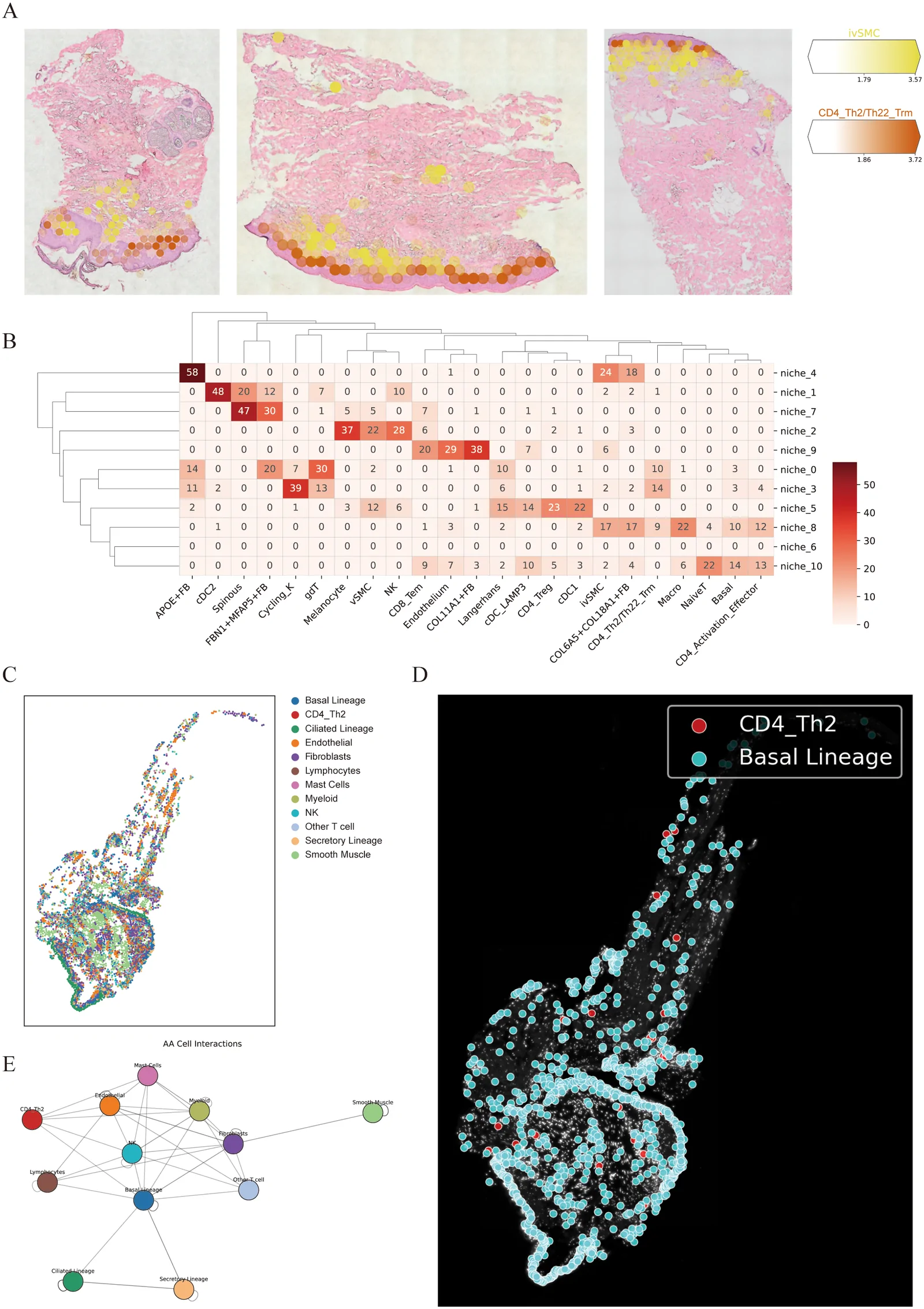
Spatial co-localization of pathogenic subclusters and effector T cells in AD and AA spatial transcriptomics. **(A)** Spatial distribution plots of ivSMC and Th2/Th22 T_rm_ cells in AD spatial transcriptomics data (cell2location deconvolution). **(B)** Heatmap of NMF spatial niche clustering. **(C)** Spatial distribution plot of cell type annotations for AA Xenium spatial transcriptomics data. **(D)** Spatial distribution plots of Basal Lineage and Th2 cells in AA tissue. **(E)** Spatial co-localization network plot of AA cells calculated based on intercellular distances. AD, atopic dermatitis; AA, allergic asthma; ivSMC, inflammatory vascular smooth muscle cell; T_rm_, tissue-resident memory T cells; NMF, non-negative matrix factorization.

Using single-cell RNA sequencing, we discovered that the pathogenic subpopulation highly expresses CCL26 and exhibits significantly enhanced communication with effector T cells. We then used spatial transcriptomics to determine their physical relationship and investigate their immune niche. Using the previously annotated AD single-cell transcriptomic data as a reference dataset, we deconvolved the AD spatial transcriptomics data from GSE206391 via cell2location, and the model fit well and converged (**Supplementary Figures 4A, B**). As shown in **Figure 11A** and **Supplementary Figure 4C**, ivSMCs and Th2/Th22 T_rm_ cells in AD patients are predominantly located at the periphery of the dermo-epidermal junction, with a tight physical proximity. NMF spatial niche clustering analysis in **Figure 11B** further demonstrated that ivSMCs, Th2/Th22 T_rm_ cells, COL6A5+COL18A1+ FBs, and macrophages in AD patient tissues exhibit distinct spatial co-localization, constituting a pathological microenvironment (Niche_8). To clarify the spatial positional relationship between the pathogenic subpopulation and Th2 cells in AA, we used marker genes from the previously described single-cell sequencing data to annotate the Xenium dataset GSE269354, with the annotation results shown in **Figure 11C**; similarly, **Figure 11D** reveals that the pathogenic subpopulation, Basal Lineage, is proximal to Th2 cells. By calculating distances between neighboring cells, we counted and normalized statistical frequencies to generate a spatial co-localization network interaction graph (**Figure 11E**), in which the Basal Lineage is directly connected to Th2 cells, demonstrating an interactive relationship.

### 3.10 *In vitro* validation of CCL26 inducibility in tissue-specific cellular models

**Figure 12:**
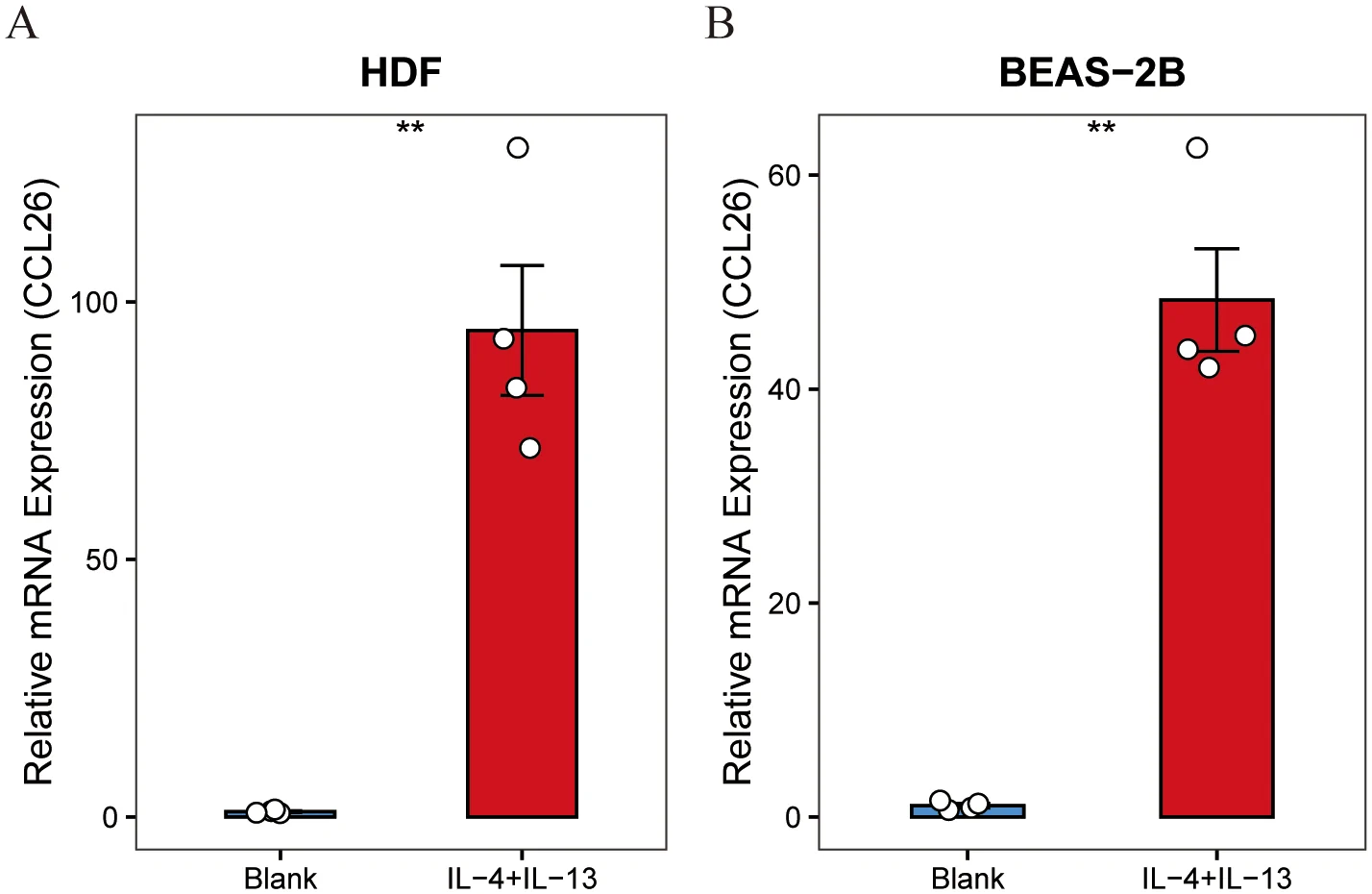
*In vitro* validation of CCL26 mRNA expression in tissue-specific cellular models following cytokine stimulation. **(A, B)** Relative mRNA expression levels of CCL26 in HDF **(A)** and BEAS-2B **(B)** cells following IL-4 and IL-13 stimulation compared to untreated controls. (**P < 0.01 vs. Blank). HDF, Human dermal fibroblasts; BEAS-2B, human bronchial epithelial cells.

Based on pseudo-bulk analysis, CCL26 was significantly upregulated in fibroblast subsets (APOE+, COL6A5+COL18A1+, and FBN1+MFAP5+ FBs) and ivSMCs in AD skin, as well as in basal and secretory lineage epithelial cells in AA airways. Given that ivSMCs represent a disease-induced *in vivo* transitional state sharing characteristic fibroblast-like markers (POSTN, COL18A1), we used HDF and BEAS-2B cells to model the cutaneous dermal mesenchyme and airway mucosal epithelium *in vitro*. Cells were treated with a combination of recombinant human IL-4 (20 ng/mL) and IL-13 (20 ng/mL) for 24 h to mimic the canonical Type 2 inflammatory microenvironment and to validate the response of the core comorbid gene CCL26 under Type 2 inflammatory conditions. Consistent with the bioinformatics findings, CCL26 expression was significantly increased in the IL-4 + IL-13 group compared with the control group in both cell types (**Figures 12A, B**). This confirms that CCL26 is upregulated in both diseases, supporting its potential as a comorbid biomarker.

## 4 Discussion

AD and AA share a type 2 inflammatory mechanism, manifesting as distinct organ-level diseases driven by a common immune environment. While systemic Th2 hyperactivation underlying their comorbidity has been reported (26), the precise molecular mechanisms connecting these two diseases at the tissue and cellular levels remain unexplored, and specific genes linking skin and airway pathologies have not been identified. In this study, we integrated multiple transcriptomics datasets, screened them using machine learning, and validated the candidates using external datasets, *in vitro* experiments, and single-cell transcriptomics data; among them, only CCL26 maintained stable and consistent expression. This cross-disease and cross-cohort stability suggests that CCL26 serves as a core gene linking the comorbidity of AD and AA. Recently, Liang et al. highlighted that shared comorbid genes across epithelial tissues (such as CDC7, PXDN, TCN1, and TIMP1) directly drive intrinsic epithelial barrier dysfunction across asthma and AD (27). Complementing these barrier-regulatory signatures, our identification of CCL26 captures stromal–immune crosstalk that recruits infiltrating effector cells across mucosal and cutaneous environments, providing a new perspective on shared atopic pathogenesis.

AD and asthma both have multiple different subtypes. By restricting asthma to IgE-mediated allergic asthma and using multiple gene-filtering methods, our study ensures the identified genes reflect genuine cross-disease comorbid biological patterns, rather than merely the consequences of Type 2 immune activation. Indeed, Sitarik et al. recently demonstrated in 5,314 children that distinct AD phenotypes have different associations with subsequent allergic diseases, yet all are associated with asthma alongside a coordinated elevation of Type 2 biomarkers (28). Furthermore, a transcriptomic study of 1,233 children by Lemonnier et al. showed that allergic multimorbidity possesses unique molecular signatures distinct from single diseases, exhibiting synergistic gene expression effects (29). Our findings extend these observations by identifying CCL26 as a comorbid gene characterized by disease-specific cellular origins and spatial inflammatory niches in the skin and airways.

CCL26, also known as eotaxin-3, is a member of the eosinophil chemotactic factor subfamily and primarily exerts a chemotactic effect on eosinophils (30). Multiple studies have confirmed that CCL26 expression is significantly elevated in the lesions and serum of AD patients (31–33), whereas after treatment with dupilumab or tralokinumab, CCL26 expression in AD lesions decreases significantly (34, 35). The International Eczema Council, using the GRADE evidence rating, classified CCL26 as moderate evidence for an AD lesion biomarker for assessing AD severity (36). Takeshi Nakahara et al., in a multicenter biomarker-cohort study, likewise found that CCL26 is the most effective biomarker for assessing Eczema Area and Severity Index severity in AD patients (37). CCL26 has also been detected in various pathological products of asthma patients (sputum, bronchoalveolar lavage fluid, biopsy, and blood) (38, 39) and serves as a biomarker for asthma onset and prevention (40). Millie Nguyen Basu et al. collected plasma from pediatric patients with AD, AA, and AD comorbid with AA, and found that CCL26 in the plasma of children with AD combined with AA was significantly higher than that in children with AD alone (41), providing direct clinical evidence that CCL26 is specifically elevated in the comorbidity state. The results obtained from our construction of nomogram calibration models for AD and AA regarding CCL26 are consistent with these clinical lines of evidence; furthermore, the Geneformer deep learning model framework further demonstrated the pathogenic effects of CCL26 in AD and AA.

By integrating results from transcriptomic sequencing, cell culture, and clinical serum assays across multiple studies to construct a chemokine network, Zahra Ahmadi et al. found that CCL26 is not merely a bystander of allergic reactions in AA and AD, but rather a core regulator directly driving the onset, development, and even exacerbation of AD and AA, while orchestrating the chronic progression of these diseases (42). Our single-cell analysis further indicated that CCL26 is not uniformly expressed across all cell types but is instead concentrated within distinct pathogenic subpopulations in both diseases.

In the AA dataset, the core pathogenic subpopulations expressing CCL26 are the basal and secretory lineages of airway epithelial cells, which are linked to CCL26’s involvement in airway remodeling and its promotion of fibroblast migration in the asthma environment (43). In T2-driven asthma, although tissue eosinophils are suppressed by high-dose inhaled glucocorticoids, abundant sputum CCL26 indicates that the airway epithelium remains in a state of inflammation and remodeling (44). Building on this, *in silico* virtual knockout of CCL26 showed that it can directly affect the IL2- and TNFα-related inflammatory pathways in AA. In addition, the upstream transcription factor TEAD4, identified by pySCENIC, is a key transcription factor for YAP/TAZ binding in the Hippo pathway, which promotes fibroblast activation and collagen deposition, leading to the hyperproliferation of airway smooth muscle and driving airway hyperresponsiveness in asthma (45); the knockout of TEAD4 in CellOracle reversed the alterations in the basal lineage and secretory lineage.

Furthermore, through the vector field flow changes in CellOracle, we discovered that AD exhibits tissue remodeling similar to that of the AA airway; CCL26 can alter the state of vSMCs in AD, causing them to present a fibrotic and inflammatory phenotype. These fibrotic ivSMCs, along with the pathogenic fibroblast subpopulations FBN1+MFAP5+ FB and APOE+ FB proposed by Helen He et al. (25), significantly overexpress CCL26 and jointly participate in the pathogenic process of AD. Virtual knockout of CCL26 in AD affected CXCL12, CXCL14, COL6A1, and TNC, which are involved in stromal cell recruitment, extracellular matrix remodeling, and tissue fibrosis, and was accompanied by reorganization of the EMT pathway.

CCR3 is a high-affinity G-protein-coupled receptor for CCL26, primarily expressed on the surface of eosinophils and effector T cells (46). Through CellChat and multinichenetr, we found that CCL26-CCR3 communication intensity between pathogenic cell subpopulations highly expressing CCL26 and Th2 cells increased in both AD and AA. In AD, ivSMCs are the primary drivers of CCL26 signaling in both healthy skin and disease states, and this output is further enhanced in AD-lesional tissues, with multiple ligand-receptor pairs linking ivSMCs and Th2/Th22 T_rm_ cells significantly elevated. In AA, the basal lineage becomes the predominant source of CCL26 signaling in the disease state, echoing the transcription factor TEAD4 identified by pySCENIC within the basal lineage. Spatial transcriptomics further confirmed interactions between these pathogenic subpopulations and Th2 cells in the skin (10x Visium) and the airway (Xenium), forming an immune microenvironment that establishes a self-amplifying inflammatory loop. In AD skin, this pathogenic crosstalk primarily engages tissue-resident Th2/Th22 T_rm_ within the superficial dermal niche (Niche_8), explaining the chronicity and persistent local recurrence of cutaneous Type 2/Type 22 inflammation.

In addition to T-cell crosstalk, the CCL26–CCR3 axis is a key driver of eosinophil recruitment and activation in AD and AA. Serum CCL26 levels are significantly elevated in AD patients and strongly correlate with SCORAD scores, peripheral blood eosinophil counts, and Th2 chemokines, highlighting its unique role in reflecting disease activity (47). In AD skin, IL-4/IL-13-stimulated fibroblasts and vascular cells produce high levels of CCL26 to recruit CCR3+ eosinophils (42), which degranulate to release cytotoxic proteins, reactive oxygen species, and TGF-β, driving epidermal barrier disruption and tissue remodeling. In AA airways, CCL26 is one of the most potent eosinophil chemoattractants. IL-13 selectively upregulates bronchial epithelial CCL26, and sputum CCL26 levels correlate closely with sputum eosinophilia, particularly in severe allergic asthma (48). Th2 cytokines induce epithelial CCL26 through the STAT6 pathway, and recruited eosinophils release additional Th2 cytokines to further promote CCL26 production and airway remodeling (49). The CCL26–eosinophil axis may also provide a mechanistic basis for AD–AA comorbidity. However, due to technical limitations of single-cell sequencing, eosinophils are easily lost during capture; future studies could use to further investigate CCL26-mediated eosinophil infiltration and activation in atopic comorbidities.

Since HaCaT is a cell line widely used for *in vitro* models of atopic dermatitis, we also investigated CCL26 induction in HaCaT cells. Compared with HDF and BEAS-2B cells, HaCaT cells exhibited the greatest change in CCL26 expression upon IL-4 + IL-13 stimulation (**Supplementary Figure 5**). However, our pseudo-bulk analysis did not identify CCL26 as a DEG within the keratinocyte cluster in AD skin. This suggests that despite the pronounced *in vitro* inducibility of keratinocytes in classical models, they may not be the primary *in vivo* source of CCL26 in AD lesions. This discrepancy may be due to the fact that Type 2 cytokine-producing cells are mainly distributed in the superficial dermis (**Figure 11A**), where resident fibroblasts are exposed to high concentrations of cytokines, whereas keratinocytes are spatially separated from cutaneous inflammatory infiltrates and encounter cytokine levels *in vivo* that are far lower than the induction concentrations *in vitro*.

Several limitations remain in the present study. First, regarding the transcriptomic data, although the sample size was expanded through cohort merging and batch-effect correction, alongside cross-validation, prospective clinical cohorts with larger sample sizes—particularly those involving patients concurrently suffering from both AD and AA—are still required for further validation to improve the diagnostic efficacy of the model. Second, our *in vitro* validation utilized two immortalized cell lines, HDF and BEAS-2B, to simulate the Type 2 inflammatory milieu via IL-4 and IL-13 stimulation, which cannot fully recapitulate the intricate immune microenvironment of the skin–airway comorbid state. In our single-cell and spatial transcriptomic analyses, ivSMCs exhibited high disease scores and prominent CCL26 expression; however, they represent an *in vivo* inflammatory transitional state with pronounced fibrogenic/fibroblast-like features. Although we functionally investigated ivSMCs through *in silico* perturbations and spatial co-localization analyses, future studies should explore direct *in vitro* modeling of the primary perivascular smooth muscle fibrogenic transition using 3D co-culture or organoid systems. Third, although *CCL26* emerged as a core comorbid mediator, direct functional validation of its causal role remains lacking. Our exploration of downstream effects and upstream transcription factors (RARB and TEAD4) relied primarily on computational *in silico* perturbation tools (CellOracle and scTenifoldKnk). We have not yet performed direct experimental validations—such as gene knockout, antibody neutralization, or *in vivo* functional assays in combined AD–AA animal models—to unequivocally establish causality. Finally, the present study focuses on the mRNA level, whereas the ultimate activation of genes and pathological remodeling are also subject to epigenetic modifications and post-transcriptional regulation; hence, a joint analysis incorporating proteomics and epigenomics was not performed.

## 5 Conclusions

This study integrated transcriptomic, machine-learning, and single-cell/spatial analyses to identify and validate CCL26 as a core comorbid gene linking AD and AA. A diagnostic model built from 175 machine-learning combinations showed strong performance across internal and external cohorts, and CCL26 upregulation was confirmed by *in vitro* IL-4 and IL-13 stimulation, with consistency across diseases and cohorts exceeding that of other candidates. Single-cell analysis localized CCL26 to specific pathogenic subclusters—ivSMCs and fibroblasts in AD skin, and basal/secretory epithelial cells in AA airways. Virtual knockout analyses linked CCL26 to tissue remodeling, extracellular matrix reorganization, and inflammatory signaling, while cell-cell communication and spatial transcriptomics showed that CCL26-CCR3 signaling connects these subclusters to effector T cells, sustaining a self-amplifying inflammatory loop in both organs—providing cross-organ molecular evidence for the atopic march. Overall, these findings establish CCL26 as a diagnostic biomarker and potential therapeutic target for AD-AA comorbidity, although its causal role still needs to be confirmed in prospective cohorts or animal models.

## Data Availability

The original contributions presented in the study are publicly available. Publicly available datasets used in this study can be downloaded from the GEO database (http://www.ncbi.nlm.nih.gov/geo/). The GSE121212, GSE130588, GSE120721, GSE16161, GSE36842, GSE32924, GSE222840, and GSE206391 were included in the AD group. The GSE41861, GSE19187, GSE193816, and GSE269354 were included in the AA group. Further inquiries can be directed to the corresponding authors.
