# Supplementary material for: Identification and experimental validation of CCL26 as a core comorbid gene and biomarker for atopic dermatitis and allergic asthma via machine learning and multi-omics analysis
Source: Front Immunol. 2026 Sep 9;17:1936742. doi: 10.3389/fimmu.2026.1936742 (PMC13597448; doi:10.3389/fimmu.2026.1936742)
Supplement: Supplementary file 1 [file Supplementaryfile1.docx]

Supplementary Material

# 1 Supplementary Figure

Fig.S1 Differential expression of nine candidate genes in the AD cohort. (A) Differential expression of nine candidate genes in the AD discovery cohort (GSE12121/GSE130588). (B) Differential expression of nine candidate genes in the AD discovery cohort (GSE121212/GSE130588). (B) Differential expression of nine candidate genes in the AD test cohort (GSE120721). (C) Differential expression of nine candidate genes in the AD test cohort (GSE16161). (D) Differential expression of nine candidate genes in the AD test cohort (GSE36842). Abbreviations: AA, allergic asthma; AD, atopic dermatitis

Fig.S2 Quality control and cellular feature distribution of the scRNA-seq dataset. (A) Violin plots showing the distribution of the number of unique genes detected per cell (*nFeature_RNA*) and the total number of RNA molecules counted per cell (*nCount_RNA*) across all sequenced samples in AD dataset. (B) Violin plots showing the distribution of the number of unique genes detected per cell (*nFeature_RNA*) and the total number of RNA molecules counted per cell (*nCount_RNA*) across all sequenced samples in AA dataset. (C) GSEA score curves for the ivSMCs subgroup in the AD dataset. (D) The UMAP plot shows the identification of six distinct cell types within the T cell subclusters from the AA dataset, accompanied by a dot plot illustrating the expression patterns of specific marker genes across each cell type.

Fig.S3 Volcano plot of differentially expressed genes in high-risk cell subclusters. (A–I) represent the ivSMC (A), vSMC (B), BEC (C), APOE+FB (D), COL6A5+COL18A1+FB (E), FBN1+MFAP5+FB (F), Basal Lineage (G), Secretory Lineage (H), and Ciliated (I) subclusters, respectively. Abbreviations: ivSMC: inflammatory vascular smooth muscle cell, vSMC: vascular smooth muscle cell, BEC: blood vascular endothelial cell, FB: fibroblast.

Fig.S4 Model evaluation and spatial distribution of cell deconvolution via cell2location. (A) The cell2location quality control plot shows the reconstruction accuracy between the observed data and posterior expected values. (B) The cell2location training history curve shows the convergence of the loss across training epochs. (C) Spatial distribution plots of ivSMC and Th2/Th22 T_rm_ cells in AD spatial transcriptomics data (cell2location deconvolution). Abbreviations: AD, atopic dermatitis; AA, allergic asthma; ivSMC, inflammatory vascular smooth muscle cell; T_rm_, tissue-resident memory T cells.

Fig.S5 *In vitro* validation of CCL26 mRNA expression in tissue-specific cellular models following cytokine stimulation. Relative mRNA expression levels of CCL26 in HaCaT cells following IL-4 and IL-13 stimulation compared to untreated controls. (*P* < 0.05, **P* < 0.01, ***P* < 0.001, ****P* < 0.0001; ns, no statistical significance). Abbreviations: HaCaT, human immortalized keratinocytes.
